# Tracing Eukaryotic Ribosome Biogenesis Factors Into the Archaeal Domain Sheds Light on the Evolution of Functional Complexity

**DOI:** 10.3389/fmicb.2021.739000

**Published:** 2021-09-16

**Authors:** Mehmet Birikmen, Katherine E. Bohnsack, Vinh Tran, Sharvari Somayaji, Markus T. Bohnsack, Ingo Ebersberger

**Affiliations:** ^1^Applied Bioinformatics Group, Institute of Cell Biology and Neuroscience, Goethe University Frankfurt, Frankfurt, Germany; ^2^Department of Molecular Biology, University Medical Center Göttingen, Göttingen, Germany; ^3^Göttingen Center for Molecular Biosciences, Georg-August University, Göttingen, Germany; ^4^Senckenberg Biodiversity and Climate Research Center (S-BIK-F), Frankfurt, Germany; ^5^LOEWE Center for Translational Biodiversity Genomics (LOEWE-TBG), Frankfurt, Germany

**Keywords:** domain architecture evolution, asgard group, phylogenetic profiles, orthology assignment, evolutionary traceability, pathway complexity, large subunit maturation

## Abstract

Ribosome assembly is an essential and carefully choreographed cellular process. In eukaryotes, several 100 proteins, distributed across the nucleolus, nucleus, and cytoplasm, co-ordinate the step-wise assembly of four ribosomal RNAs (rRNAs) and approximately 80 ribosomal proteins (RPs) into the mature ribosomal subunits. Due to the inherent complexity of the assembly process, functional studies identifying ribosome biogenesis factors and, more importantly, their precise functions and interplay are confined to a few and very well-established model organisms. Although best characterized in yeast (*Saccharomyces cerevisiae*), emerging links to disease and the discovery of additional layers of regulation have recently encouraged deeper analysis of the pathway in human cells. In archaea, ribosome biogenesis is less well-understood. However, their simpler sub-cellular structure should allow a less elaborated assembly procedure, potentially providing insights into the functional essentials of ribosome biogenesis that evolved long before the diversification of archaea and eukaryotes. Here, we use a comprehensive phylogenetic profiling setup, integrating targeted ortholog searches with automated scoring of protein domain architecture similarities and an assessment of when search sensitivity becomes limiting, to trace 301 curated eukaryotic ribosome biogenesis factors across 982 taxa spanning the tree of life and including 727 archaea. We show that both factor loss and lineage-specific modifications of factor function modulate ribosome biogenesis, and we highlight that limited sensitivity of the ortholog search can confound evolutionary conclusions. Projecting into the archaeal domain, we find that only few factors are consistently present across the analyzed taxa, and lineage-specific loss is common. While members of the Asgard group are not special with respect to their inventory of ribosome biogenesis factors (RBFs), they unite the highest number of orthologs to eukaryotic RBFs in one taxon. Using large ribosomal subunit maturation as an example, we demonstrate that archaea pursue a simplified version of the corresponding steps in eukaryotes. Much of the complexity of this process evolved on the eukaryotic lineage by the duplication of ribosomal proteins and their subsequent functional diversification into ribosome biogenesis factors. This highlights that studying ribosome biogenesis in archaea provides fundamental information also for understanding the process in eukaryotes.

## Introduction

Due to the essential role of ribosomes in producing all cellular proteins, their synthesis is among the few pathways that are universally necessary for organismic life. Despite substantial differences in the ways that the ribosomal proteins (RPs) and ribosomal RNAs (rRNAs) are assembled into the small and large ribosomal subunits (SSU and LSU, respectively), the fundamental basis of this process in the three domains of life (Woese et al., 1990) presumably dates back to the last universal common ancestor (LUCA). Ribosome assembly is most simple and best understood in bacteria. Bacterial ribosomes can be assembled *in vitro* without the requirement for any non-ribosomal proteins while up to approximately 30 assembly factors make the process much faster and more accurate *in vivo* (Shajani et al., 2011). In contrast, the functional network mediating the same process in eukaryotes is many-fold more extensive, and it is to date best studied in yeast (*Saccharomyces cerevisiae*; Hage and Tollervey, 2004; Henras et al., 2008; Sloan et al., 2017; Klinge and Woolford, 2019). A recent study listed 255 ribosome biogenesis factors with a confirmed or suspected direct role in this process (Ebersberger et al., 2014). Assigning these factors to different age layers revealed that precisely how eukaryotes mediate ribosome assembly is not cast into stone. Instead, an evolutionarily old set of core functions, whose emergence predates the diversification of eukaryotes, was extended and probably fine-tuned in a lineage-specific manner (Ebersberger et al., 2014). Archaea seem to assume an intermediate position between eukaryotes and bacteria with respect to the complexity of their ribosome assembly pathways (Lecompte et al., 2002; Londei and Ferreira-Cerca, 2021). Shifting focus toward the archaeal domain therefore has the potential to further disentangle the building principles of ribosomes that already evolved prior to the emergence of eukaryotes and were already established in the last eukaryotic common ancestor (LECA). Thus far, phylogenetic profiling indicated that 38 yeast RBFs have counterparts in the archaeal domain (Ebersberger et al., 2014), in parts representing functional sub-clusters that seem specifically involved in the late steps of ribosome maturation. A subset of these factors has been subsequently confirmed as RBFs by functional studies in individual archaeal models (reviewed in Londei and Ferreira-Cerca, 2021). However, we are far from fully comprehending the extent to which archaeal ribosome biogenesis resembles that of eukaryotes, where archaea have implemented alternative strategies, and the degree of diversity within the archaeal domain.

Much of the uncertainty about the common grounds of ribosome biogenesis in archaea and eukaryotes is connected to methodological issues in the large-scale profiling studies performed thus far. The objective of such studies is easily specified: “Identify the functionally equivalent archaeal protein to a eukaryotic RBF, if it is present.” Its realization, however, bears numerous pitfalls. Unidirectional searches for sequences with a significant local similarity e.g., with BLAST (Altschul et al., 2009) or Diamond (Buchfink et al., 2015) rapidly identify archaeal RBF candidates at a high sensitivity, however, the specificity is low (Chen et al., 2007). Considering only such proteins as RBF candidates that diverged no longer than the species they reside in (orthologs; e.g., Koonin et al., 2001; Ebersberger et al., 2014) reduces the false positive rate, because orthologs are the best guesses when searching for functionally equivalent proteins across species (Tatusov et al., 1997; Altenhoff et al., 2012). However, the higher specificity comes at the cost of a decrease in sensitivity (Altenhoff et al., 2016). Moreover, also orthologs may have diverged in function (Ebersberger et al., 2014). The latter problem can be ameliorated by scoring the similarity of protein domain architectures (Koestler et al., 2010), which comprise features, such as Pfam (El-Gebali et al., 2019) or Smart (Letunic et al., 2009) domains, transmembrane regions, signal peptides, or regions of biased amino acid composition. Differences in domain architectures can then indicate alterations in the respective functional spectra of the compared proteins (Jiang et al., 2020). In contrast to the ample means to cope with the spurious inference of archaeal RBF candidates based on homologous proteins, false negatives, i.e., proteins overlooked in the homolog searches because their sequences have diverged to an extent that they are no more similar than it is expected by chance, have received very little attention. Such proteins can represent missing links that are essential for concluding on the presence of functional (sub-)network based on phylogenetic profiling analyses (Jain et al., 2019). Their identification requires a targeted increase of the search sensitivity accompanied by a careful downstream curation to validate the results.

Next to the methodological issues outlined above, at least three further aspects leave the current understanding of common concepts in eukaryotic and archaeal ribosome biogenesis incomplete. In recent years, numerous yeast proteins have been discovered as RBFs (e.g., Fujiyama-Nakamura et al., 2009; van Tran et al., 2019) whose presence in the archaea has not been tested in a comprehensive screen, thus far. Moreover, structure- and mass spectrometry-based approaches have shed light on the order of selected events during the assembly process and the identity of the participating proteins (see for example, Wu et al., 2016; Barandun et al., 2017; Kater et al., 2017; Sanghai et al., 2018; Klingauf-Nerurkar et al., 2020; Liang et al., 2020; Nieto et al., 2020). This resource, which provides an excellent basis for identifying functional sub-networks shared between eukaryotes and archaea, is largely untapped. Moreover, the focus on yeast, a highly derived model organism that lost many genes essential in other species (Peter et al., 2018), is a limiting factor itself. Knowledge on the yeast RBFs is now complemented by large-scale, RNAi-based screens in human cells, which have revealed several 100 proteins that may be either directly or indirectly required for human ribosome biogenesis (Wild et al., 2010; Tafforeau et al., 2013; Badertscher et al., 2015; Farley-Barnes et al., 2018). For a non-negligible fraction of these RBF candidates a yeast homolog is elusive (Fujiyama-Nakamura et al., 2009; Tafforeau et al., 2013; van Tran et al., 2019; Ameismeier et al., 2020). It is conceivable that at least some of these factors act as RBFs in archaea too. Lastly, both the number of archaeal taxa and their phylogenetic diversity in the public sequence databases has dramatically increased in the past few years. This data provides an excellent, yet largely unused, basis for a highly-resolved analysis of the representation of eukaryotic RBFs in the archaeal domain. Among others, it allows to test whether members of the Asgard group share more RBFs with the eukaryotes than other archaea, which would be in line with their suspected placement as the closest relative of the eukaryotes (Zaremba-Niedzwiedzka et al., 2017; Imachi et al., 2020; Liu et al., 2021).

Here, we re-address the evolutionary history of eukaryotic ribosome biogenesis and trace the deep evolutionary roots of this pathway that are shared with the archaea. We base our analysis on a set of 301 manually curated yeast and human RBFs comprising the, to date, most comprehensive collection of eukaryotic ribosome biogenesis factors. Phylogenetic profiles for each protein across more than 900 taxa, among them 727 archaea including representatives of the Asgard group, using targeted and domain architecture aware ortholog searches provide insights into the evolution of this pathway at an unprecedented resolution. The analysis is complemented by identifying RBFs that evolve too quickly to facilitate ortholog identification over longer evolutionary time scales. This helps reconciling the discrepancy between large-scale phylogenetic profiling of RBFs using ortholog/homology assignments claiming the absence of an RBF and experimental evidences showing the presence of the corresponding function. In the same line, it can direct the attention to missing functional links that may require searches at higher sensitivity to warrant their detection.

## Materials and Methods

### Selection of Ribosome Biogenesis Factors

We compiled an initial non-redundant set of 307 yeast proteins putatively involved into ribosome biogenesis. About 255 RBF candidates were obtained from Ebersberger et al. (2014), 41 from the KEGG Brite database (KO3009; Kanehisa et al., 2016), and additionally 11 from recent publications focusing on molecular details of yeast ribosome biogenesis. The candidate list together with the references is given in Supplementary Table S1. For the human RBF collection, we seeded the set with 198 proteins that are involved into human ribosome biogenesis according to KEGG (Kanehisa et al., 2016). This data were complemented with 488 proteins with at least a suspected involvement into human ribosome biogenesis according to large scale screening studies (40S/60S: Wild et al., 2010; pre-rRNA processing: Tafforeau et al., 2013; 40S: Badertscher et al., 2015; and regulators: Farley-Barnes et al., 2018). Finally, we added eight RBFs that emerged from a literature screen (Yang et al., 2006; Freed et al., 2012; Wandrey et al., 2015; van Tran et al., 2019; Ameismeier et al., 2020). The non-redundant list of 695 proteins excluding ribosomal proteins is provided in Supplementary Table S2. The phylogenetic profiles of all human candidates were determined and served as basis to assess their evolutionary age (see below).

### Taxon Collection

We determined the phylogenetic profiles for the RBF candidates across 982 taxa comprising 232 eukaryotes, a diverse collection of 23 bacteria representing 16 phyla, and 727 archaea. Archaeal gene sets were retrieved from the RefSeq partition of the NCBI Genome database (O’Leary et al., 2016; December 2020). The 78 Quest for Orthologs reference proteomes^1^ complemented with 189 fungal taxa served as representatives for the bacterial and eukaryotic taxa. The taxon list is provided in Supplementary Table S3.

### Pathway Analysis

Information about pathway organization and complex composition was obtained from KEGG (Kanehisa et al., 2021) and Reactome v76 (Fabregat et al., 2018).

### Domain-Aware Phylogenetic Profiling and Gene Age Estimation

RBF orthologs were identified with the targeted ortholog search tool fDog (Jiang et al., 2020)^2^ using the following parameter settings: –checkCoorthologsRef, −-countercheck, −-minDist=family, and −-maxDist=kingdom. Protein domain architectures were compared pair-wise between each seed protein and its orthologs, and an architecture similarity score was computed with FAS^3^ implemented into fDOG. In brief, FAS compares the domain architectures of two proteins, using one architecture as the reference and the second architecture as the query. The FAS score ranges between 0 (architectures are completely dissimilar) and 1 (the architecture of the reference is at least a sub-architecture of the query; Koestler et al., 2010). Because the score is not symmetric, we computed FAS scores once using the seed protein as reference (FAS forward), and once using the ortholog as reference (FAS backward). The Domain architecture-aware phylogenetic profiles generated by fDog were visualized and analyzed with PhyloProfile (Tran et al., 2018). The evolutionary emergence of individual RBF candidates was dated using an LCA algorithm implemented into PhyloProfile. In brief, the last common ancestor (LCA) of the two most distantly related species harboring an ortholog to an RBF candidate was used as a minimal age estimate for the corresponding gene. Comparisons of FAS score distributions and domain architecture comparisons between two taxonomic groups were performed with the group comparison function implemented into PhyloProfile.

### Computation of Evolutionary Traceability

Orthologs of quickly evolving proteins may lose a sufficiently high sequence similarity to warrant their detection already over short evolutionary distances. The evolutionary traceability index (Ti) is a simulation-based score on the interval [0,1] that captures, for a protein and a given evolutionary distance, whether an ortholog likely still shares a sufficiently high sequence similarity that allows its detection, or whether it likely has diverged beyond recognition (Jain et al., 2019). We extracted, for each yeast protein, the corresponding traceability indices across 273 taxa distributed across the tree of life from Jain et al. (2019). Traceability indices on a kingdom level were computed as the mean Ti across all members in the kingdom. An RBF was considered as low traceable in case the mean traceability index fell below 0.75 (Jain et al., 2019). Note, we limited this computation to the yeast proteins, since the human RBFs were pre-selected based on the presence of orthologs already in evolutionary distantly related taxa.

### Phylogenetic Analyses

Multiple Sequence alignments were generated with Muscle v3.8.1551 (Edgar, 2004) using default settings. Sequence logos were generated with WebLogo (Crooks et al., 2004) provided as a web service available at https://weblogo.berkeley.edu. For maximum likelihood phylogenetic tree reconstruction with IQ-TREE using the LG+G+I model of sequence evolution (Nguyen et al., 2015), alignments were post-processed by removing alignment columns with more than 50% gaps. Phylogenetic trees were visualized with ITol (Letunic and Bork, 2021).

## Results

### Ribosome Biogenesis From Two Perspectives

**Figure 1:**
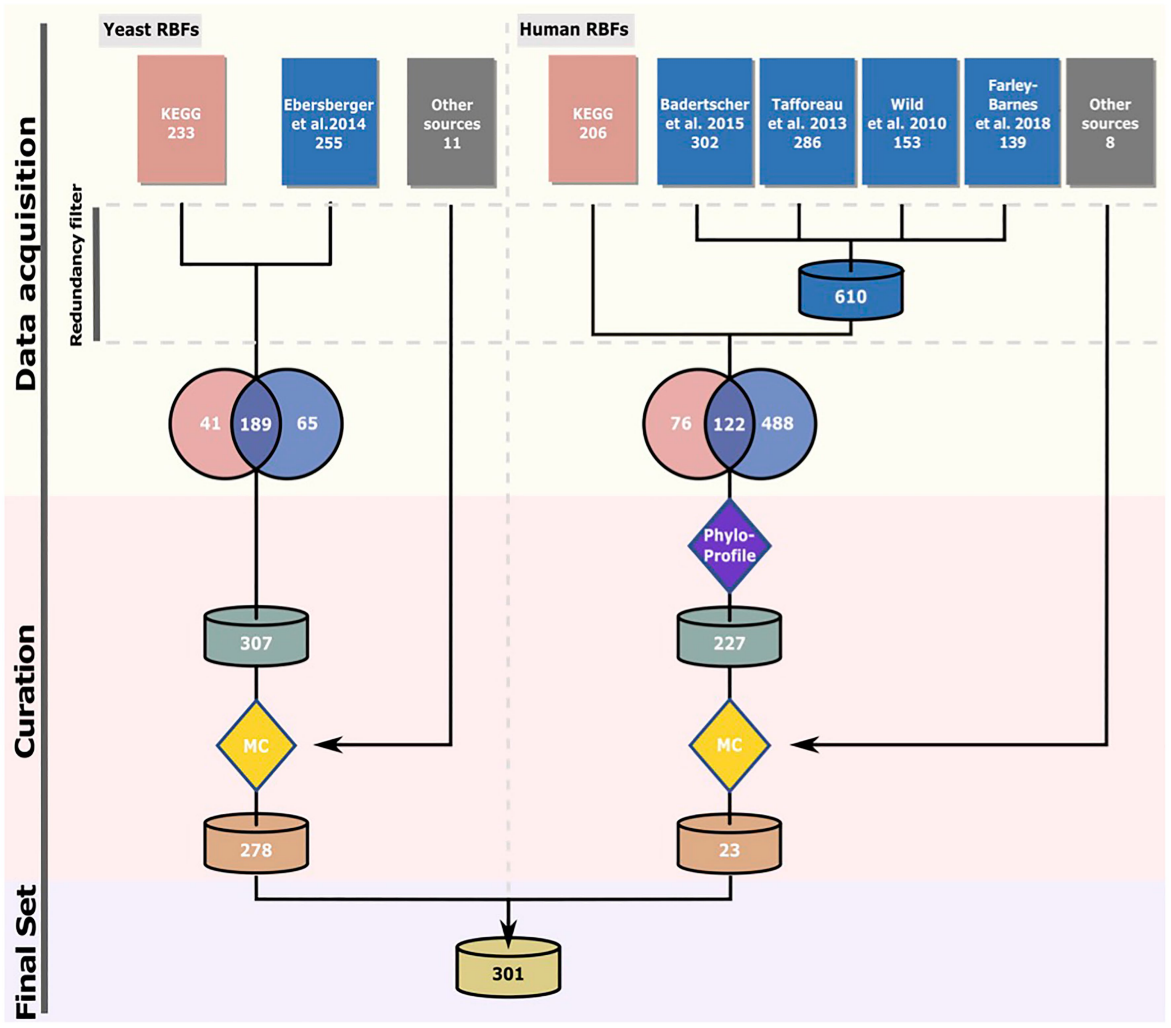
Compilation of the RBF set. The “Phylostratigraphy” filter removed proteins younger than last eukaryotic common ancestor (LECA) and proteins with an ortholog in yeast. In the manual curation (MC) step, only proteins for which experimental evidence supports their association with pre-ribosomal particles or requirement for ribosome assembly were retained. See text for details. The sources for the candidate proteins are specified in Supplementary Table S1 (yeast) and Supplementary Table S2 (human).

Production of ribosomes is essential for organismic life. Here, we set out to identify the common basis of ribosome biogenesis in eukaryotes and archaea using a manually curated list of eukaryotic RBFs (Figure 1). The most comprehensive inventory of protein factors involved in eukaryotic ribosome biogenesis is that of the yeast *S. cerevisiae* S288C (Woolford and Baserga, 2013; Bassler and Hurt, 2019). An initial screen for yeast RBFs retrieved 307 candidates (see Figure 1 for numbers and references). To consider also eukaryotic RBFs that are either absent in yeast or have a function other than RBF, we used an initial set of 686 potential human RBFs obtained largely from comprehensive RNAi-based screens (Wild et al., 2010; Tafforeau et al., 2013; Badertscher et al., 2015; Farley-Barnes et al., 2018) as a second starting point of the analysis.

The phylogenetic profile for each RBF candidate was subsequently determined by searching for orthologs in 232 eukaryotes, 727 archaea, and 23 bacteria. For each detected ortholog, we then computed the domain architecture similarity to that of the corresponding yeast or human seed protein. On this basis, a two-stage curation procedure was devised to extract the final set of RBF candidates for further analysis. In the evolutionary-motivated first stage (Supplementary Figure S1; Supplementary Table S4), the subset of RBFs that can be traced back to the LCA of all eukaryotes (LECA) was identified. We then removed all proteins with a yeast ortholog that is considered already in the yeast RBF collection leaving 227 human proteins. We implemented this first stage for the human RBF candidates to reduce the number of proteins that enter the second stage of manual curation. In the subsequent curation step, we retained only proteins that fulfill at least one of the following criteria: (i) have a known function in ribosome biogenesis; (ii) have been identified associated with pre-ribosomal particles; or (iii) their depletion or deletion causes a defect in ribosome assembly. This retained 278 yeast proteins (RBF_yeast_) and 17 human proteins (RBF_human_). We complemented this data with six known human RBFs, ILF2, ILF3, TMA16, NOL11, NKRF, and ZCCHC4, most likely younger than the LECA. The final set, RBF_euk_, comprised 301 factors. We added two subunits of the RNA polymerase II, Rpb5, and Rpo21, as positive controls for our profiling approach. The two proteins are evolutionarily highly conserved and orthologs should be identifiable in the majority of the species analyzed here. Thus, the total number of analyzed proteins sums up to 303.

### PhyloRBF: Interactive Access to the Data

**Figure 2:**
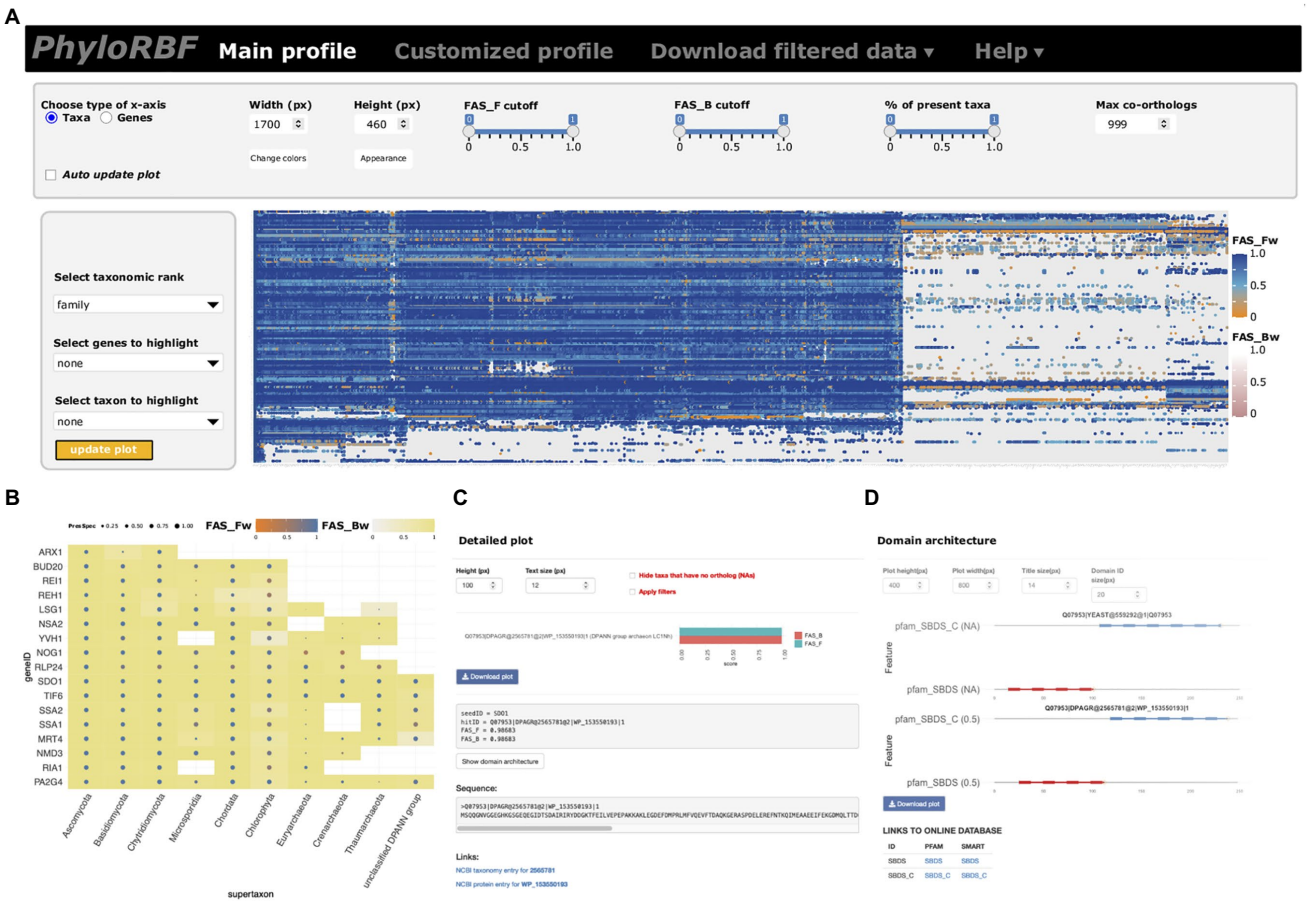
The PhyloRBF platform. **(A)** Data overview; **(B)** Custom selection of genes and proteins for display; **(C)** Detail information for seed-ortholog pairs; and **(D)** Domain architecture view.

The phylogenetic profiles for proteins in the RBF_euk_ set across 982 taxa provide the first unifying resource for tracing eukaryotic ribosome biogenesis factors across the organismal diversity. To facilitate easy and interactive exploration and analysis of these data, we provide two options. An online instance of PhyloProfile (Tran et al., 2018), PhyloRBF, provides web-access to these data *via* the URL: https://applbio.biologie.uni-frankfurt.de/phylorbf. Users can display the full data set (Figure 2A), customized subsets of RBFs and taxa (Figure 2B), zoom in on individual ortholog pairs (Figure 2C), and ultimately display the domain architectures of the yeast or human RBF and of its ortholog (Figure 2D). Interactive links connect the information about taxon, protein sequence and Pfam (El-Gebali et al., 2019), or SMART domains (Letunic et al., 2009) with the corresponding public databases. For an offline analysis of the RBF_euk_ phylogenetic profiles with a local installation of PhyloProfile, the input data are provided as Supplementary Data 2.

### The Evolutionary Trajectory of Eukaryotic Ribosome Biogenesis Factors

**Figure 3:**
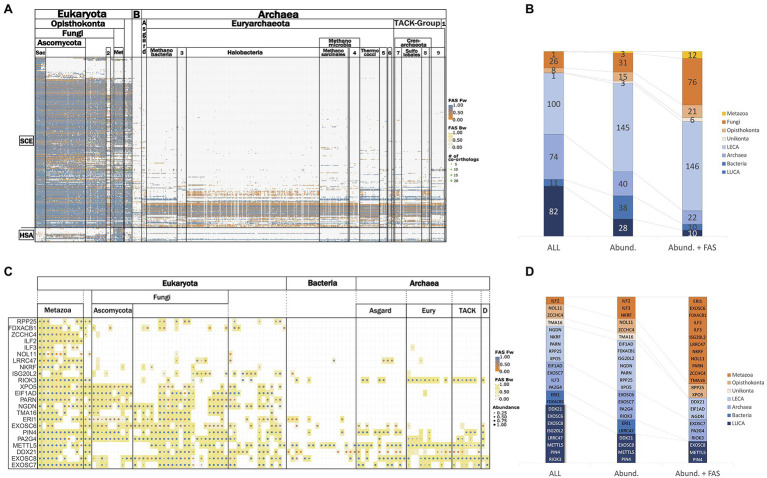
Representation of RBF_euk_ orthologs across the three domains of life. **(A)** Phylogenetic profile of the RBF_euk_ set summarized on the class-level. Proteins are represented by the rows and columns represent the analyzed taxa. A dot in the matrix indicates that an ortholog for the respective RBF was found in this taxon. The dot color informs about the domain architecture similarity between the detected ortholog and the seed protein. **(B)** Phylostratigraphy of the RBF_euk_ set based on the data shown in **(A)** filtered at varying levels of stringency (see main text). LECA – last eukaryotic common ancestor; Archaea – orthologs to an RBF are present in the archaea but not in the bacteria; Bacteria – orthologs to an RBF are present in bacteria but not in archaea; and last universal common ancestor (LUCA) – an ortholog was present both in archaea and bacteria. The two positive controls are placed in the Archaea stratum for the All and the Abundance filter, but are placed in LECA in the Abund. + FAS filter. **(C)** Phylogenetic profiles of the RBF_human_ set summarized on the class level. The corresponding phylostratigraphies are shown in **(D)**.

Figure 3A provides an overview of the phylogenetic profiles for the RBF_euk_ set and the two positive controls. Within eukaryotes, orthologs of most RBFs are represented in all investigated lineages. Losses of RBFs are rare, but nevertheless they seem to occur. Examples exist of yeast RBFs lacking orthologs in either all or at least most of the Pezizomycotina, the sister clade of the Saccharomycotina, although orthologs are found in more distantly related fungi (Supplementary Figure S2). Substantially more pronounced is the apparent loss of RBFs in the microsporidia, obligate intracellular fungal parasites, which lack orthologs to 56 yeast RBFs that can be traced back to LECA (Supplementary Table S4). This reveals that the overall trend of microsporidia to evolve toward a highly simplified variant of a eukaryotic organism (Keeling et al., 2010) extends also to ribosome biogenesis. Likewise, 17 human RBFs are represented in a diverse set of eukaryotes, but orthologs are missing either in all fungi, or specifically on the lineage toward *S. cerevisiae* (see below).

In contrast to the overall conservation of RBFs in the eukaryotic domain, the representation of orthologs in archaea and bacteria is substantially sparser. Bacteria lack orthologs for most the eukaryotic RBFs, and only individual factors are detected consistently across the sampled taxa. The picture in archaea is more differentiated; archaea possess orthologs of a considerable number of RBFs. For individual factors, orthologs are consistently detected across the individual archaeal groups, whereas for others, they are confined to individual lineages or they occur only sporadically. Overall, very few factors are ubiquitously present across all three domains of life or are consistently present in the bacterial representatives but not in the archaea.

To provide a more quantitative view of these observations, we stratified the proteins into different age layers. In the most permissive setting (Figures 3B–D), we assessed the minimal evolutionary age of a protein by assigning it to the LCA that the seed species shares with the most distantly related species in which an ortholog was detected. However, such unfiltered data may suffer from overestimates due to spurious orthology assignments and gene sets contaminated with sequences from other species (Steinegger and Salzberg, 2020). We therefore introduced two filters to reconstruct the phylostrata with two increasing levels of confidence (see Supplementary Figure S3 for a visualization of the filtering effect). An abundance filter was established, building on the observation that secondary losses of RBFs are rare. It is, thus, expected that an RBF predicted to be present in the LCA of a systematic group, should be detected in all, or at least the majority, of its descendants. In turn, orthologs that show low prevalence in a group are likely spurious or represent contaminations that can be disregarded. For the second filter, we additionally propose that functionally equivalent orthologs participating in ribosome biogenesis in different organisms should have similar domain architectures (Koestler et al., 2010). We, thus, applied the abundance filter on the phylogenetic profiles where we kept only orthologs with a domain architecture that is similar to that of the seed protein (delta-FAS<0.25). The resulting phylostratigraphies after application of the filters are shown in Figure 3B, and the corresponding assignment of the RBFs to the individual phylostrata is provided as Supplementary Table S5. As expected, the proportion of RBFs assigned to evolutionarily younger phylostrata increases with filtering strength, and even the two positive controls were assigned only to LECA in the very stringent combined abundance and FAS filter. The results for the human RBFs are shown in Figures 3C,D. We note, however, that an *ad hoc* specification of the appropriate filtering criteria is difficult. The phylostratigraphies shown in Figure 3 should be interpreted such that they allow dynamic analysis strategies. The most stringent filtering serves to reconstruct the evolutionary scaffold of ribosome biogenesis across the tree of life at high confidence. This scaffold can then be successively extended, where appropriate, with results obtained only with the more permissive filters. This clearly indicates where a focus on subsequent data curation and/or experimental validation should be placed.

### Absence of Human RBFs in the Fungal Lineage – Functional Plasticity or Methodological Artefact?

Figure 3A reveals that evidence of lineage-specific losses of RBFs within the eukaryotes is rare, with the notable exception of the microsporidia. This makes the existence of 17 human RBFs that can be traced back at least to LECA, but either lack an ortholog in yeast, or their ortholog has diverged in function, an intriguing observation. Analyses of these proteins over, in the scope of this study, the moderate evolutionary distances between human and yeast serves two important needs. They can highlight that even evolutionarily old mechanisms of eukaryotic ribosome biogenesis can change on individual lineages. Probably even more important, they can shed light on methodological issues that become relevant when extending the analysis to the substantially more distantly related archaea.

METTL5 and PIN4 (Parvulin 14) represent two particularly prominent examples of gene loss on the evolutionary lineage leading to *S. cerevisiae*. Both proteins are assigned to the evolutionarily oldest RBF stratum under all filtering conditions (*cf.* Figures 3C,D). Within fungi, METTL5 is confined to the early branching lineages with only three putative orthologs in the Basidiomycota. METTL5 is an RNA methyltransferase that mediates *N*^6^-methylation of adenosine 1832 in the 18S rRNA in humans (van Tran et al., 2019), and a similar function has been described for METTL5 in *Haloferx vulcanii*, where it installs m^6^A1432 in the 16S rRNA (Kowalak et al., 2000; Grosjean et al., 2008). Yeast, in turn, has no m^6^A present at the corresponding position of the 18S rRNA (Taoka et al., 2016; Sergiev et al., 2018). The loss of METTL5 in the course of fungal diversification is, thus, an evolutionarily unique event that changed a long-standing event in ribosome biogenesis. PIN4, a peptidyl-prolyl cis-trans isomerase required for pre-rRNA processing (Fujiyama-Nakamura et al., 2009), displays a different timing of gene loss. Like METTL5, PIN4 is also present in the archaea (Figures 3C,D), which is in line with previous findings (Jaremko et al., 2011; Hoppstock et al., 2016). In contrast to METTL5, PIN4 is prevalent in fungi. A sequence comparison between fungal and animal PIN4 orthologs indicates the presence of the sequence motif relevant for the association of this protein with pre-ribosomal complexes (Supplementary Figure S4). The loss of PIN4 immediately predates the diversification of the Saccharomycetales. The functional consequences of this loss are, to our knowledge, not yet explored. It will be interesting to see whether yeast and its close relatives utilize a non-homologous protein to functionally replace PIN4, whether its action is not necessary in the context of yeast ribosome biogenesis, or whether an alternative mechanism exists to compensate this loss of function.

### Domain Architecture Changes Indicate Lineage-Specific Loss of RBF Function

**Figure 4:**
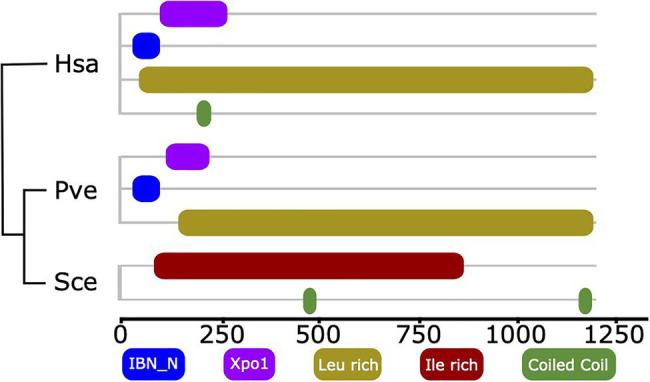
Domain architecture comparison of Xpo5 between human and fungal orthologs. The two Pfam domains linked to import/export activity of human Xpo5, IBN_N (Pfam ID: PF03810) and Xpo1 (Pfam ID: PF08389) are still present in an early branching fungus and were secondarily lost in yeast. The phylogenetic relationships of the three taxa are indicated by the tree. Hsa, *Homo sapiens*; Pve, *Podila verticillata* (Mucoromycota); and Sce, *Saccharomyces cerevisiae*.

The example of XPO5 reveals that events subtler than gene loss also need to be considered when tracing factors involved in ribosome biogenesis across the organismic diversity. XPO5 is a human export factor that shuttles a diverse set of cargos, most prominently non-coding RNAs, such as tRNAs and pre-miRNA, but also ribosomal subunit precursors out of the nucleus (Bohnsack et al., 2002; Calado et al., 2002; Yi et al., 2003; Bohnsack et al., 2004; Lund et al., 2004; Wild et al., 2010; Leisegang et al., 2012). The corresponding ortholog in yeast serves also as an export factor, but with a more restricted set of cargoes that does not include ribosomal subunits (Moy and Silver, 1999; Stage-Zimmermann et al., 2000; Sloan et al., 2016). Notably, the domain architectures of human XPO5 and its ortholog in yeast, Msn5, are substantially different (Figure 4). Two N-terminal Pfam domains, Xpo1 (Pfam ID: PF08389) and IBN_N (Pfam ID: PF03810), that are characteristic for human XPO5 and also other proteins involved in nuclear import and export, e.g., yeast Xpo1 (P30822), are missing in yeast Msn5. Notably, we find the human domain architecture for XPO5 reflected in orthologs from early branching fungal lineages, suggesting that here the contribution of this protein to ribosome biogenesis could still be maintained. Although experimental proof that the apparent loss of the two domains causes the shift in the functional spectrum of Msn5 has to delivered, this result suggests that changes in domain architecture can highlight RBF orthologs that may have altered their function.

### Evolutionary Traceability of RBFs

**Figure 5:**
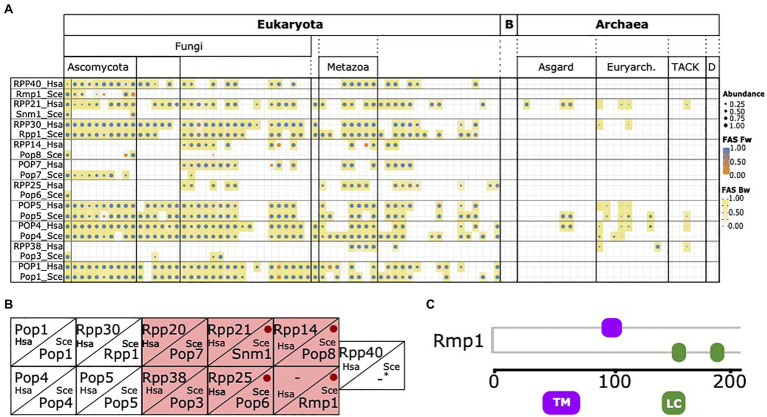
The phylogenetic profile of the RNase MRP components. **(A)** The phylogenetic profiles of the 10 yeast and human RNase MRP components. Human and yeast components considered functionally equivalent are placed in one box. **(B)** Corresponding components of RNase MRP complex in yeast and human according to Rosenblad et al. (2006). Yeast proteins with a missed human ortholog in **(A)** are highlighted in red. Red dots indicate yeast proteins with a traceability index below 0.75 in humans, indicating that orthologs most likely have diverged beyond recognition (Snm1=0,58; Pop6=0,48; Pop8=0,58; and Rmp1=0). For human RPP40 and yeast Rmp1 no corresponding protein in the other species are known. **(C)** Domain architecture of yeast Rmp1. TM, transmembrane domain; LC, low complexity region. See Supplementary Figure S4 for the domain architectures of the remaining five proteins.

During integration of the RBF_yeast_ and RBF_human_ sets, no human protein was considered that has a yeast ortholog also annotated as an RBF. Interestingly, we noted that individual human factors, in particular, six of the 10 protein components of the RNase MRP complex, were initially retained because no yeast ortholog could be detected (Figure 5A). While this is consistent with the notion that yeast has substantially modified its RNase MRP complex because orthologs of many of its components are confined to yeast and its close relatives (Ebersberger et al., 2014), it is at odds with experimental evidence that functional equivalents exist for these factors in yeast and the function of the RNase MRP complex in pre-rRNA processing is conserved between yeast and humans (Chu et al., 1994; Lygerou et al., 1996; Rosenblad et al., 2006; Goldfarb and Cech, 2017; Figure 5B). To resolve this contradiction, we first confirmed that the absence of a yeast ortholog is independently supported by the InParanoid database (Ostlund et al., 2010), one of the most sensitive and specific public databases for ortholog assignments between pairs of species (Altenhoff et al., 2016). Thus, the missing of orthologs for these proteins in the respective species seems inherent to the ortholog search itself, and is not dependent on the ortholog search tool. Investigating the six proteins in greater detail revealed that all are short with lengths between 150 and 200 amino acids, and Rmp1 and Pop8 are additionally devoid of any Pfam domain (Figure 5C; Supplementary Figure S5). Both characteristics in combination are indicative for proteins that lose a significant sequence similarity to their orthologs already over small evolutionary distances (Jain et al., 2019). Indeed, the evolutionary traceability indices for these proteins in humans (see Materials and Methods) indicate that human orthologs of four proteins likely have diverged beyond recognition (*cf.* Figure 3B), and thus escape detection in large-scale phylogenetic profiling studies. In conclusion, the discrepancy between the *in silico* approaches to assess phylogenetic distribution and evolutionary age of the yeast RNase MRP components, and the experimental evidence, can be explained in at least four out of six cases by the limited sensitivity of the ortholog search.

**Figure 6:**
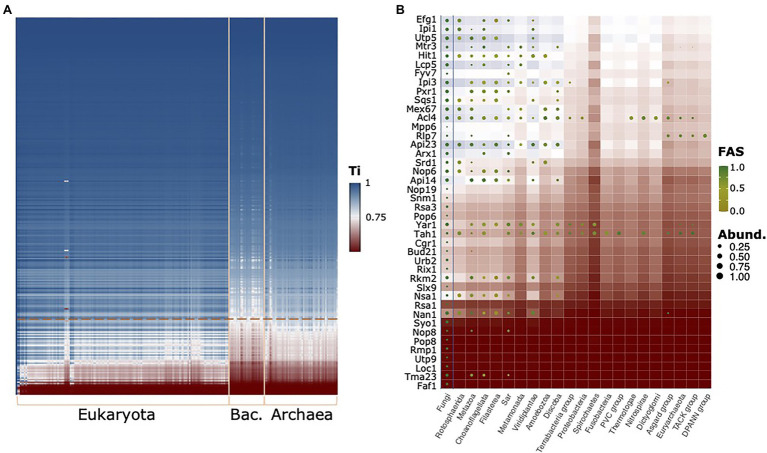
The evolutionary traceability of the yeast RBFs. **(A)** The heat map reveals proteins and taxa for which the ortholog search sensitivity likely becomes a limiting factor (white to red areas). The phylogenetic profiles for proteins with a mean kingdom-wide traceability index (Ti) below 0.75 are shown in **(B)** summarized on a kingdom level. Abund – Fraction of taxa subsumed in a kingdom harboring an ortholog; FAS – maximum domain-architecture similarity between a yeast protein and the orthologs subsumed in this group. Background color represents the evolutionary traceability for the respective yeast protein.

To see whether other proteins in the RBF_euk_ set may suffer from the same limitations, in particular, when extending the scope of the ortholog search to the archaeal domain, we subsequently computed the traceability indices across the full range of taxa considered here (Figure 6A). This identified 42 proteins that evolve at rates likely to hinder detection of orthologs in evolutionarily more distant lineages (Figure 6B). For these proteins, it is important to consider that the phylogenetic profiles may underestimate the taxonomic range in which orthologs are present, and hence will result in underestimated evolutionary ages. Among them, we find a further factor, Arx1, which associates with late LSU particles in the nucleus and facilitates their export (Bradatsch et al., 2007; Hung et al., 2008). PA2G4, which is a member of the RBF_human_ set, was initially suggested to be its functional equivalent in humans, but we failed to establish orthology relationships between Arx1 and PA2G4 (Supplementary Figure S6). Meanwhile, evidences accumulated that PA2G4 differs in regions that are implicated in nucleoporin interaction in Arx1 (Squatrito et al., 2004; Bradatsch et al., 2007; Wild et al., 2010; Bhaskar et al., 2021). While this suggests a functional diversification between the two proteins, their precise evolutionary relationships remain unclear. A phylogenetic analysis revealed gene duplication paired with a substantial acceleration of the evolutionary rate on the Arx1 lineage of Saccharomycotina and Taphrinomycotina (Supplementary Figure S7). This lends support to the hypothesis that similar to the components of the RNase MRP complex, the orthology between PA2G4 and Arx1 was overlooked thus far.

### Eukaryotic RBFs in the Archaeal Domain

The analysis thus far has set the methodological stage for projecting concepts of eukaryotic ribosome biogenesis into the archaeal domain. Subsequently, we focused on the representation of the RBF_euk_ in archaea. Sampling more than 700 taxa covering the full known archaeal diversity, including the Asgard group that are proposed as the closest relatives to the eukaryotes (Zaremba-Niedzwiedzka et al., 2017), constitutes an unprecedented basis for this analysis. Most importantly, it provides the first comprehensive overview of which factors are represented by orthologs in the archaeal domain, together with information about their lineage-specific prevalence (Supplementary Figure S8). For 156 factors in the RBF_euk_ set, we found in the unfiltered data at least one ortholog in the archaeal domain (*cf.* Figure 3B; Supplementary Figure S8). The number of orthologs per species varies considerably (Supplementary Table S5), and *Candidatus Prometheoarchaeum syntrophicum* (Asgard group) harbors the highest number of RBF orthologs (67) across the sampled taxa. Applying an abundance filter (see above) to select only proteins consistently seen in the archaea reduces the number to 17 factors that are represented by an ortholog in more than 85% of the investigated taxa (Supplementary Table S7), among the two positive controls. This number reduces to only four when setting the inclusion threshold at 90%. These nearly ubiquitously represented proteins comprise two RNA methyltransferases, METTL5, and Nop1/Fibrillarin, the pseudouridine synthase Pus7 that catalyzes pseudouridylation within the 5S rRNA, and one of our two positive controls, the largest subunit of the RNA polymerase II, Rpo21. The presence of many of these proteins, and particular of these latter factors, in archaea is already well acknowledged (e.g., Jaremko et al., 2011; Ebersberger et al., 2014; Londei and Ferreira-Cerca, 2021). Their prevalence in our data set suggests that insights gained from detailed experimental analyses in individual taxa can, in some cases, be projected to the entire archaeal domain. Factors that are present only in a subset of the taxa, in turn, can provide insights into lineage-specific differences of presumably related to ribosome biogenesis within the archaea, an area that is still considerably uncharted.

### Lineage-Specific Presence of Eukaryotic RBFs in Archaea

**Figure 7:**
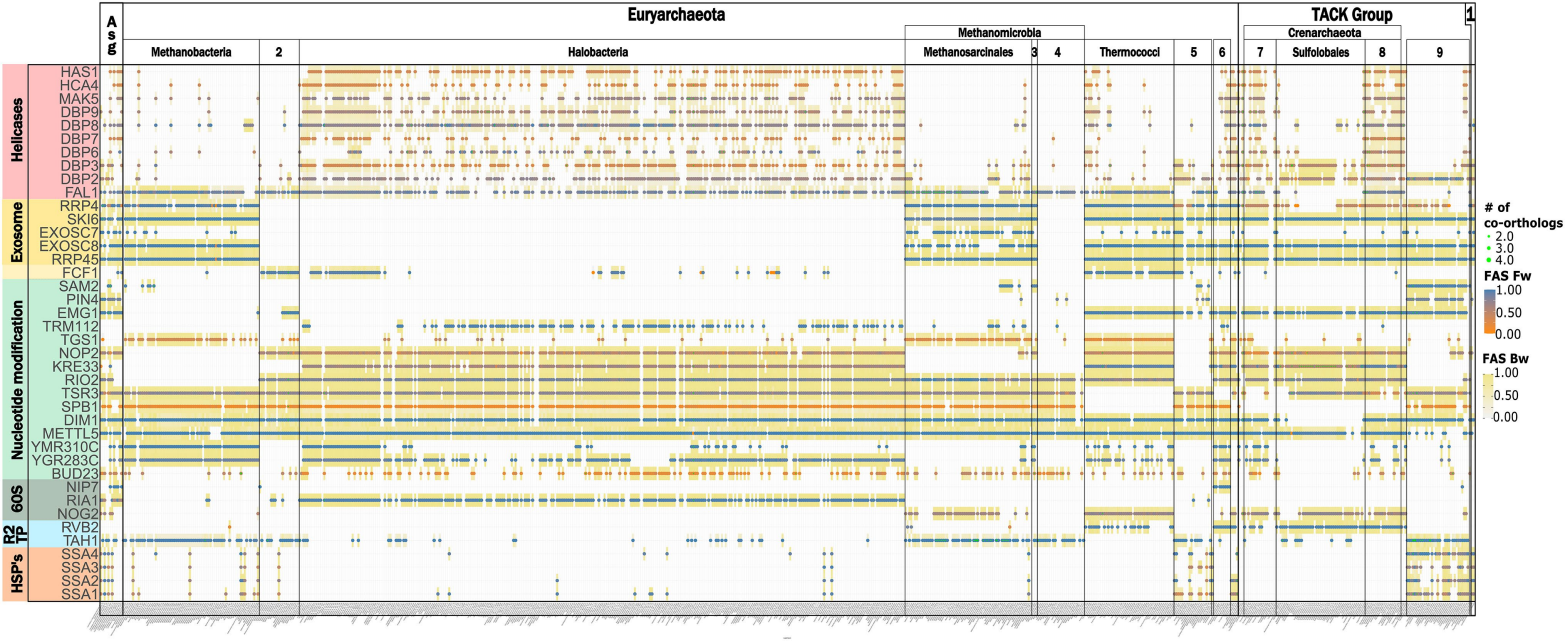
The differential presence-absence pattern of eukaryotic RBFs in the archaeal domain summarized on the class-level. 1: DPANN, 2: Methanococci, 3: Methanocellales, 4: Methanomicrobiales, 5: Thermoproteales, 6: Archaeoglobi, 7: Thermoproteii, 8: Desulfurococcales, and 9 Thaumarchaeota.

From the archaeal section of Figure 3A, we selected the subset of RBF_euk_ that is confined to individual archaeal clades and grouped these proteins into seven functional categories (Figure 7). This revealed a loss of several components of the RNA exosome, a major RNA decay machinery (Sloan et al., 2012), once in Halobacteria and once in Methanomicrobiales. Two small-scale analyses hinted toward the possible absence of the exosome complex in these clades (Koonin et al., 2001; Phung et al., 2020). The concerted absence of the exosome components across all members of the two clades seen here, provides substantial evidence that the RNA exosome has indeed been lost on these lineages. A similarly prominent signal is seen for the RNA helicases. However, we note that the domain architecture similarities between the RBFs and their archaeal orthologs are typically small for these proteins (Figure 7; Supplementary Figure S9). As RNA helicases are involved in a plethora of different processes in a cell, it is thus too early to conclude on the existence of these RBFs in the archaeal domain. The other functional categories do not share a consistent phylogenetic profile. The HSP70-family chaperones SSA1-4 are no exception here, as they all share the same archaeal proteins as orthologs, indicating that gene duplications on the eukaryote lineage gave rise to these four distinct RBFs. This directs further attention to the presence/absence pattern of individual proteins, most of them involved in nucleotide modification. Here, the missing of orthologs to Dim1, a dimethyladenosine transferase involved in rRNA modification in the Sulfolobales is, at first glance, an intriguing finding. Dim1 is a protein that is otherwise almost fully conserved across taxon collection. We detected an ortholog in 99% of the eukaryotic taxa and it is present in all but one (*Aquifex aeolicus*) of the bacterial species. The domain architectures of both eukaryotic and bacterial/archaeal orthologs are virtually identical to that of yeast Dim1 (Supplementary Figure S10), and the traceability of Dim1 is one across all taxa. Despite this convincing indication for a gene loss, the finding is at odds with the detection of an *N*^6^-dimethyladenosine in the 16S rRNA (Noon et al., 1998) and a recent characterization of a Dim1-like enzyme in *Sulfolobus acidocaldarius* (Knuppel et al., 2021). In the first step, we confirmed that a BlastP search with the alleged Dim1 from *S. acidocaldarius* revealed no hit in yeast (not shown). To increase the sensitivity of the ortholog search, we used a “stepping-stone” approach. In brief, we performed a second search for Dim1 orthologs, this time seeding the search with the Dim1 of *S. acidocaldarius*. This served to reduce the evolutionary distance between seed protein and possible orthologs in the archaea (Martin-Duran et al., 2017). This search identified orthologs throughout the archaeal domain and sporadically also in the eukaryotes (Supplementary Figure S11). In all investigated cases, these orthologs were the same as those obtained with yeast Dim1 as a seed sequence. Based on their overlapping phylogenetic profiles, we conclude that yeast Dim1 and the protein in *S. acidocaldaricus* are indeed orthologs, although they share no significant sequence similarity. A further comparison revealed that Dim1 in Sulfolobales is only about 200 aa in length, while Dim1 in other taxa has a length of around 300 amino acids (Supplementary Figure S12). In summary, one of the evolutionarily most conserved proteins known to date has been modified in one archaeal lineage comprising largely extremophiles, such that the orthologs have diverged, from a eukaryotic viewpoint, beyond recognition. It will be interesting to see the precise functional consequences that are likely accompanied with this change. In contrast to the spurious absence of Dim1, there is strong evidence for a lineage specific loss of the kinase/ATPase Rio2, the RNA helicase/acetyltransferase Kre33, and the RNA methyltransferase Emg1 within the archaea. For these proteins, the stepping-stone approach provided no indication of overlooked orthologs (Supplementary Figure S13), and we conclude that the gaps in their phylogenetic profiles represent the genuine absence of these proteins in the respective taxa.

### Large Subunit Processing in Archaea

**Figure 8:**
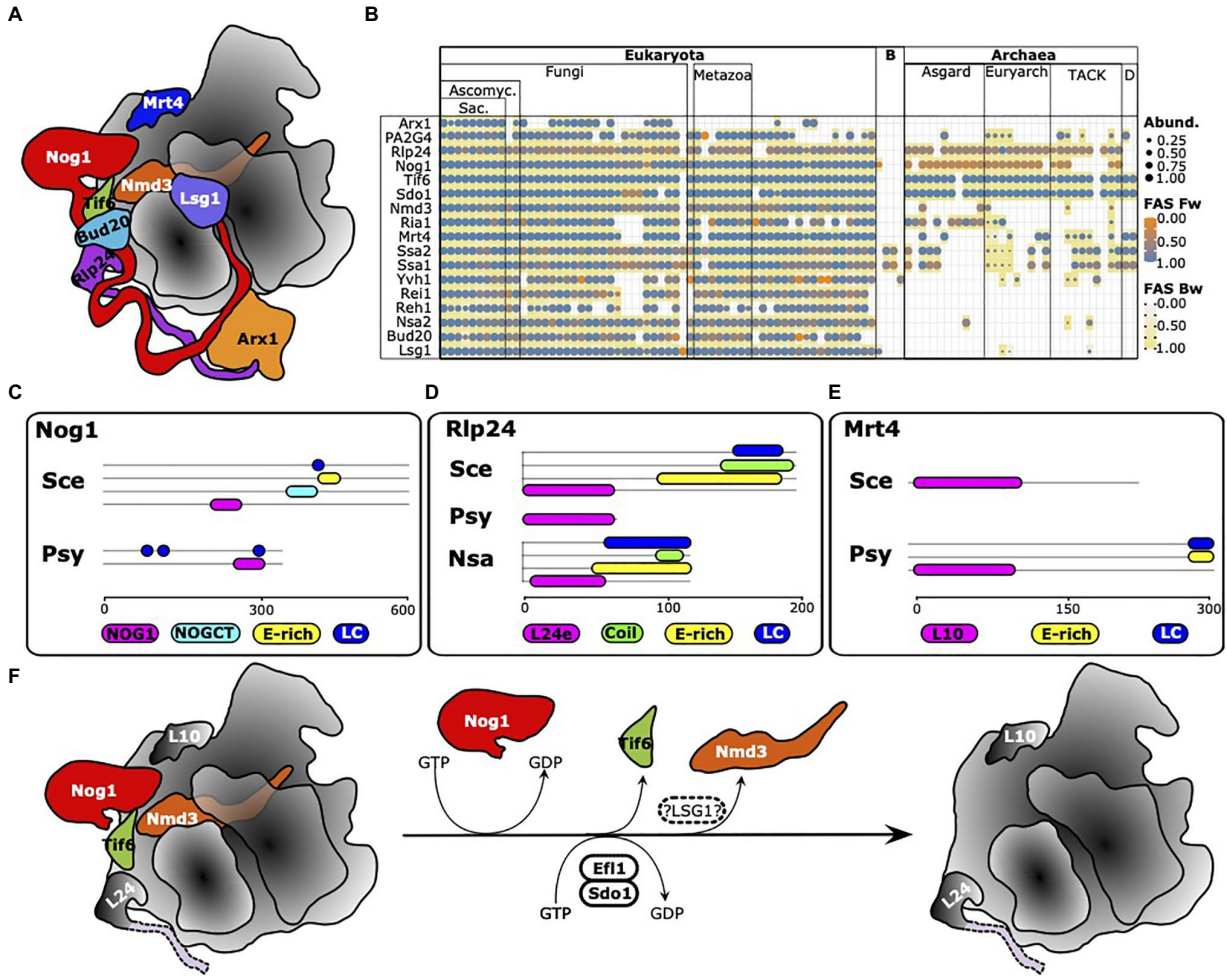
Maturation of the large ribosomal subunit in archaea is a simplified version of the process in eukaryotes. **(A)** Ribosome biogenesis factors involved in eukaryotic pre-60S maturation. Figure re-drawn from Klingauf-Nerurkar et al. (2020). **(B)** Phylogenetic profiles of the factors involved in yeast 60S maturation. PA2G4 is a human functional equivalent to Arx1 in yeast (see text for details). **(C-E)** Domain architectures of yeast Nog1, Rlp24, and Mrt4 and of a representative archaean ortholog. Sce, *S. cerevisiae*; Psy, *Prometheoarchaeum syntrophicum*; and Nsa, *Natronomonas salsuginis*. Protein ids: Nog1_Sce_ – Q02892; Nog1_Psyn_ – WP_147661298; Mrt4_Sce_ – P33201; Mrt4_Psyn_ – WP_147662854; Rlp24_Sce_ – Q07915; Rlp24_Psyn_ – WP_147664435; and Rlp24_Nsa_ – WP_137275029. **(F)** Essential steps of large subunit maturation likely to be conserved between eukaryotes and archaea. Thus far, we have no evidence for the presence of LSG1 in archaea (hatched oval).

The analyses thus far resulted in a high-resolution overview of the prevalence and distribution of eukaryotic RBFs in the archaeal domain. However, they also revealed that a face-value interpretation of phylogentic profiles can be misleading with the erroneous assumption of factor (and function) absence and presence. Careful curation of the profiles *via* a comparison of domain architectures, the consideration of the evolutionary traceability and case-by-case, more sensitive analyses *via* the stepping-stone approach, or even *via* the search for proteins harboring only a characteristic Pfam domain (see Jain et al., 2019), can substantially increase the reliability of the conclusions drawn. Such comprehensive analyses are challenging to apply on large data sets and are performed best in a context of functional sub-clusters. Here, we focused on late steps during maturation of the 60S subunit regulated by the GTPase Nog1 (Klingauf-Nerurkar et al., 2020) as a showcase example (Figure 8A). The phylogenetic profiles of 16 RBFs involved are shown in Figure 8B and reveal an intriguing pattern. Only two factors are consistently present in the archaea with domain architecture similarities that leave little doubt of functional equivalence to the yeast proteins, Tif6 (Benelli et al., 2009) and Sdo1. These proteins functionally interact, where Sdo1, in combination with Ria1 (see below), promotes removal of Tif6 from pre-60S particles. Orthologs to Nog1 are found in all major archaeal lineages, except in the two representatives of the DPANN group. However, their domain architecture similarity scores are low (Supplementary Figure S14), which indicates either a spurious ortholog assignment or a change in protein function during the evolution of Nog1. In strong support of the latter hypothesis, we first confirmed that in fungi, animals, and archaea, typically only a single gene in the gene set of an organism encodes a protein carrying a Nog1 Pfam domain (Pfam Id: PF06858; Supplementary Figure S15). A subsequent comparison of the domain architectures revealed that archaeal Nog1 is only half the size of its eukaryotic counterpart (Figure 8C). While the N-terminal part harboring the GTPase activity is present, archaeal Nog1 lacks the C-terminal portion that mediates the interaction with Arx1. The situation is similar for Rlp24, which was suggested to act as placeholder for the ribosomal protein eL24 during the maturation process (Warner, 2015; Figures 8B,D), but with two key differences. Almost all archaeal orthologs of Rlp24 are approximately 70 amino acids in length and harbor only the L24e Pfam domain (PF01246). The C-terminal tail that is essential for recruiting Drg1 is missing. But surprisingly, we find individual taxa within the Halobacteria and the Thermoplasmata whose Rlp24 ortholog is slightly longer. Their domain architectures match with that of yeast Rlp24, and they also appear in possession of the C-terminal tail of the protein found in yeast despite the absence of Drg1 orthologs in these clades. The second difference is the functional annotation of the archaeal orthologs. In all cases, we identified the ribosomal protein L24 as the ortholog to Rlp24, and this is the only protein encoded in archaeal genomes that harbors the L24e Pfam domain, again leaving no scope for overlooked orthologs (Supplementary Figure S16). This is perfectly in line with a scenario where eukaryotic Rlp24 and eL24 emerged by a gene duplication of an ancient ribosomal protein. One copy retained the ancestral function as a ribosomal protein, whereas the other copy evolved into an RBF. Interestingly, the same scenario applies to Mrt4 (Figures 3A,E), which is known to function as a placeholder for the acidic 60S ribosomal protein uL10 (Warner, 2015). The archaeal orthologs are annotated as Rpl10, and again each of the investigated archaeal taxa harbors exactly one protein with the L10e domain (Supplementary Figure S17). Notably, the archaeal orthologs can be longer than yeast Mrt4. They often harbor an acidic (E-rich) stretch and a low complexity region at the C-terminus (Supplementary Figure S18), similar to what is seen for yeast Rlp24 and Nog1 (Figures 8C,D). Judging by the domain architecture, they seem to resemble the eukaryotic ribosomal protein P0 rather than Mrt4, indicating that the architecture of Mrt4 is the evolutionarily-derived form (Supplementary Figure S19). Note, the considerably sparse representation of Mrt4 orthologs must be attributed to methodological issues (see Dim1 above), as all archaeal gene sets we checked contained exactly one protein with the L10e Pfam domain (*cf.* Supplementary Figure S17). Validating the presence of the other proteins involved in late 60S maturation in archaea provided unambiguous evidence only for Nmd3 (Supplementary Figure S20), a protein that, in eukaryotes, contributes to pre-60S nuclear export by acting as an adaptor for the nuclear transport receptor Crm1 (Ho et al., 2000). Efl1/Ria1, which interacts with Sdo1 in the release of Tif6, identifies the elongation factor EF2 in archaea as an ortholog. Similar to Mrt4 and Rlp24, also eukaryotic EF2 and Efl1/Ria1 likely emerged by a gene duplication in LECA (see Supplementary Figures S21, S22 for details). All other proteins appear absent in the archaea. In case an ortholog was identified in our search, it turned out to resemble a remote paralog of the RBF with an evolutionarily highly conserved function, e.g., MAP2 in the case of PA2G4 (*cf.* Supplementary Figure S6). A single factor, however, remains elusive, Lsg1. Its GTPase activity is required for the release of Nmd3 as one of the last steps of 60S maturation (Hedges et al., 2005). Searches at higher sensitivity identified potential homologs, but neither sequence similarity nor domain architecture conservation sufficed to conclude on the presence of this protein in the archaea. Taken together, these data suggest that many late steps of large subunit maturation in archaea follow the same principles as in eukaryotes. However, instead of using the placeholders Mrt4 and Rlp24, archaea most likely directly install the ribosomal proteins RPP0 and L24. As a consequence, the downstream machinery required for the successive release of Rlp24 and Mrt4 by Drg1 (Pertschy et al., 2007) and Yvh1 (Kemmler et al., 2009; Lo et al., 2009), and the incorporation of corresponding ribosomal proteins, is not necessary. Likewise, the lack of an ortholog to Arx1 in the archaea is in line with the findings that the C-terminus of Nog1, which is essential for interaction with Arx1, is absent from the archaeal proteins. Interestingly, Mrt4 also represents one of the factors that is implicated in pre-ribosome surveillance in eukaryotes (reviewed in Karbstein, 2013; Klinge and Woolford, 2019), as it was reported to block ribosomal stalk assembly until it is released from pre-ribosomal complexes (Kemmler et al., 2009; Lo et al., 2009; Rodriguez-Mateos et al., 2009). Its absence in archaea might therefore indicate a simplification of the process in this clade. In essence, it appears that the late steps of large subunit maturation in contemporary archaea largely resemble the primordial pathway in the LCA of archaea and eukaryotes. This basic functionality was later extended in the eukaryotic lineage to facilitate nuclear export and to implement various quality control steps.

## Discussion

Eukaryotic ribosome biogenesis is currently best understood in the yeast model system, *S. cerevisiae*, but knowledge on the human pathway is growing (Bohnsack and Bohnsack, 2019). However, the increasing availability of genome sequences of species from the remotest corners of cellular life now enables investigation of the conservation and plasticity of this pathway across the eukaryotic and the archaeal domains. Integrating these diverse efforts into a comprehensive view of how ribosome biogenesis is accomplished across the organismal diversity strongly benefits from a unifying data basis. This allows an interpretation of the outcomes of functional studies on individual RBFs or complexes contributing to ribosome biogenesis in the context of their evolutionary trajectory across the organismic diversity. Various public databases partially fulfil this requirement. For example, KEGG (Kanehisa et al., 2016) or REACTOME (Fabregat et al., 2018) provide access to pathways and interaction networks of proteins involved in ribosome biogenesis together with a representation of pathway components in other taxa. Dedicated ortholog databases, such as InParanoid (Ostlund et al., 2010), OMA (Altenhoff et al., 2018), and orthoDB (Zdobnov et al., 2017) provide comprehensive collections of orthologous groups across the full proteomes of hundreds to thousands of species. Despite the wealth of information contained in these resources, they also have limitations. Neither KEGG nor REACTOME consider the full set of factors currently considered as RBFs. Furthermore, the ortholog databases have no focus on a dedicated pathway, they provide no direct access to phylogenetic profiles for individual or groups of proteins, and they do not facilitate a comparison of domain architectures or a scoring of architecture similarities. Last but not least, it is almost impossible to extend the analysis to custom factors or species. Here, we have combined data from various resources to compile a manually curated, non-redundant set of 301 eukaryotic RBFs (*cf.* Figure 1). Their domain architecture-aware phylogenetic profiles across more than 900 taxa are a first step to close these gaps in the currently available resources. The data can be interactively explored and analyzed *via* a light-weight web-portal, or for more in-depth analyses, downloaded and analyzed offline. They can be visualized as a whole to explore the concerted evolutionary behavior of groups of proteins (*cf.* Figure 7), or subsets of taxa and proteins can be extracted for in depth analyses down to exploring the lineage-specific fate of individual protein domains (Roustan et al., 2016). This allows to discern factors whose functions likely have changed between orthologs from those where no such indication exists (Jiang et al., 2020). Eventually, the data basis is flexible. Our approach makes it straightforward to extend these profiles with further taxa or factors in custom fashion. We therefore hope that the broad community interested in ribosome biogenesis will benefit from this data and it could serve as template for further pathway-specific analyses.

### Secondary Modification of Ribosome Biogenesis in Fungi

Large-scale screens in humans paired with targeted identification of proteinaceous factors *via* the characterization of ribosomal complexes have the potential to complement the yeast perspective on eukaryotic ribosome biogenesis (Wild et al., 2010; Tafforeau et al., 2013; Badertscher et al., 2015; Farley-Barnes et al., 2018). Interestingly, the integration of this data revealed only 23 human factors that lack an ortholog in yeast. We note that this number is most likely an underestimate. Manual curation determining whether a protein was already shown to associate with pre-ribosomal particles/factors or has a known direct function in the pathway was reserved to human proteins at least as old as LECA. Thus, many candidates in the screens have simply not been analyzed in enough detail yet to know if they fulfil these criteria. Still, 17 evolutionarily old RBFs are missing in yeast and in part also in other fungal taxa. This indicates that traditional concepts of ribosome biogenesis have been modified during fungal diversification, despite the overall evolutionary conservation of this process (Wild et al., 2010; Tafforeau et al., 2013; Badertscher et al., 2015; Farley-Barnes et al., 2018; Bohnsack and Bohnsack, 2019). With METTL5 and XPO5, we have highlighted one prominent example each for loss-of-function and alteration of function, respectively. Specifically, we could date the loss of METTL5 most likely to the LCA_Dikarya_. Thus, the absence of m^6^A that has been described for yeast most likely applies to all dikarya. We could correlate a change in the domain architecture of Msn5, the yeast ortholog of XPO5, with an expansion of its functional repertoire. On closer inspection, we observed a recurrent change in domain architecture within the ascomycetes for this protein, and it will be interesting to see to what extent this affects the cargo(s) Msn5/XPO5 can transport. Few other human RBFs are absent in fungi, and the effect of their alleged loss in fungi should be investigated in greater detail and related with their functions in other eukaryotes.

### Sensitivity of the Ortholog Search as a Limiting Factor

The integration of the human data has opened up a further aspect that has so far received little attention in the interpretation of presence/absence patterns in phylogenetic profiles. A number of human factors for which functionally equivalent proteins exist in yeast, appear to lack yeast orthologs. Taken at face value, this must be interpreted as evidence for non-orthologous functional replacements (Koonin et al., 1996). However, for quickly evolving proteins, the grey zone where orthologs are no more similar than it is expected by chance (Rost, 1999; Jain et al., 2019) is within reach, even for the evolutionary distances between humans and yeast, and even more so when extending analyses into the archaeal domain. Here, we have provided evidence that for 42 yeast RBFs, the sensitivity of the ortholog search can become a limiting factor. Among these we found many proteins whose functional equivalent in humans is not identified as an ortholog, eventually reconciling the findings from targeted functional and the large-scale evolutionary approaches. Increasing the search sensitivity, e.g., by switching to unidirectional profile-based searches scanning for proteins sharing the same Pfam domains as the target protein, is an obvious solution. This, however, comes at the costs of a substantially reduced specificity making careful downstream validation of the findings necessary (Jain et al., 2019). However, the example of Dim1 shows that traceability indices can also be positively misleading. The underlying simulation approach is based on the assumption that evolutionary rates and constraints for a protein do not change over time. Our results indicate that this does not apply to Dim1 in the Sulfolobales. The lineage-specific change of both rate and mode of sequence evolution for this otherwise ubiquitously conserved protein, which causes the orthologs to be missed, is intriguing. Whether this is a consequence of an altered function of Dim1 and/or a change of the selective constraints resting on the corresponding gene remains to be determined. While experimental evidence exists for the presence of Dim1 in the Sulfolobales (Knuppel et al., 2021); this is not necessarily the case for other proteins. In such cases, the stepping-stone approach that serves to reduce the evolutionary distance over which orthologs need to be identified can be used to identify even evolutionarily diverged orthologs.

### Eukaryotic Ribosome Biogenesis Factors in the Archaeal Domain

Catalogues of eukaryotic ribosome biogenesis factors that are also present in the archaeal domain have been compiled before (e.g., Ebersberger et al., 2014). These earlier studies sufficed to identify the presence/absence of factors in individual archaeal species. Yet, the limited taxon sampling did not allow to differentiate between signal and noise with respect to varying abundance patterns across the analyzed taxa. Along the same line, the co-occurrence of eukaryotic RBFs in individual taxa, an obvious pre-requisite for but also an indication of their functional interaction, could not be exhaustively tested. Here, we could show that, in contrast to the situation in the eukaryotes, very few factors are consistently found throughout the archaeal domain. This can be an effect of limited search sensitivity (see above). However, even the application of the stepping-stone approach indicated the lineage-specific absence of otherwise essential factors, such as Kre33, Rio2, and Emg1. These findings can serve as starting points to elucidate whether ribosome biogenesis in the archaea is more plastic than is the case for the eukaryotes, whether individual factors are not essential (e.g., Knuppel et al., 2021), or whether they convey their activity to a different pathway.

Within our taxon sampling, we considered also all sequenced representatives of the Asgard group, which have been proposed to be the closest relatives to eukaryotes (Zaremba-Niedzwiedzka et al., 2017). This suggested close relationship is not reflected by the presence of any eukaryotic RBF exclusively in members of this clade. However, we note that among all analyzed archaeal taxa, the recently sequenced *Candidatus Prometheoarchaeum synthrophicum* (Asgard group) harbors the largest number of orthologs to eukaryotic RBFs (Supplementary Table S9). Whether this is co-incidence or if this indicates that ribosome biogenesis in this species is indeed more similar to that of eukaryotes remains to be determined.

### Large Subunit Maturation in Archaea

Over the past years, the structural characterization of pre-ribosomal complexes together with studies on the precise functions of the proteins involved have elucidated the roles and interplay of many RBFs in the stepwise formation and maturation of the ribosomal subunits (e.g., Saveanu et al., 2003; Woolford and Baserga, 2013; Henras et al., 2015; Wu et al., 2016; Bassler and Hurt, 2019; Bohnsack and Bohnsack, 2019; Kargas et al., 2019; Zhou et al., 2019; Klingauf-Nerurkar et al., 2020; Liang et al., 2020). Clusters of RBFs provide an unprecedented opportunity to *in silico* “assemble” archaeal RBF orthologs into a comprehensive picture. This allows well-informed and testable predictions about differences and similarities of the corresponding processes in archaea. Projecting the steps in late maturation of the 60S subunit (Kargas et al., 2019; Zhou et al., 2019; Klingauf-Nerurkar et al., 2020) into the archaeal domain revealed that archaea follow, by and large, similar principles compared to eukaryotes. Yet this process is significantly more simplistic. The absence of C-terminal extensions in both archaeal Nog1, and L24, the archaeal ortholog of both the eukaryotic RBF Rlp24 and the ribosomal protein eL24, indicates that the parts of the maturation pathway that require protein-protein interaction mediated *via* these extensions (Klingauf-Nerurkar et al., 2020) are missing in the archaea. Moreover, the ribosomal proteins uL10 and eL24 appear associated with the 50S ribosomal complex directly, without the need for previous recruitment of placeholder proteins. The apparent simplicity of this process probably contributes to the fact that the 50S subunit of various archaeal species can be spontaneously reconstituted *in vitro* with RBFs (Londei et al., 1986; Sanchez et al., 1990). The eukaryotic RBFs Mrt4 and Rlp24 that serve as placeholders for the RPPs uL10 and eL24, respectively, emerged by a duplication of the genes encoding the corresponding ribosomal proteins only in the LECA. Consequently, some factors that are required for steps leading up to the installation of these ribosomal proteins in eukaryotes were not identified in archaea and it is probably safe to consider them absent. In essence, archaea seem to bypass various assembly and surveillance steps during large subunit maturation that are characteristic for eukaryotes. Overall, this represents a showcase example of the evolution of complexity *via* the duplication and subsequent functional diversification of ancient genes (Ohno, 1970; Taylor and Raes, 2004) paired with a lineage-specific modification of evolutionary old genes.

Two aspects however remain puzzling. Firstly, the mechanism of release of the pre-60S biogenesis and export factor Nmd3, which is ubiquitously found in archaea, is elusive, as we did not find any evidence for an archaeal counterpart to the GTPase Lsg1, which releases Nmd3 from the 60S complex in yeast (Malyutin et al., 2017). While its eukaryotic function in pre-60S export will not be present in archaea due to the absence of a nucleus, archaeal Nmd3 might share its role in uL16 loading onto pre-ribosomes with its eukaryotic counterpart (Hedges et al., 2005; Hofer et al., 2007; Kargas et al., 2019; Zhou et al., 2019). Future experiments will have to elucidate the precise role of Nmd3 orthologs in the archaea and whether a different protein may serve as the functional equivalent to Lsg1. Secondly, our observation that the ribosomal protein L24 in archaea comes in two flavors is intriguing. The most widespread form is a short protein harboring exclusively the Ribosome_L24e Pfam domain. Representatives of Halobacteria and of Thermoplasmata, however, possess a protein that resembles a miniaturized version of the yeast RBF Rlp24. Their L24 includes a C-terminal extension with exactly the same features as in the yeast protein, a coiled-coil domain, an E-rich stretch and a low-complexity region. These proteins have a second interesting characteristic. Eukaryotic Rlp24 share with archaeal but not with eukaryotic L24e the presence of four conserved cysteine residues at the N-terminus (Saveanu et al., 2003). Three of the four Cysteine residues have been mutated in the L24e with the eukaryote-like domain architecture (Supplementary Figure S23). So far, we have no evolutionary explanation for these observations and it will be interesting to investigate the functional role of these changes.

In summary, we established a common data base for integrating research on ribosome biogenesis in eukaryotes and archaea. We have highlighted the potential, as well as possible pitfalls, in the interpretation of these data and have highlighted the cascade of *in silico* analyses that is necessary to faithfully reconstruct the evolutionary trajectory of individual or groups of RBFs from these data. Tracing eukaryotic RBFs into the archaeal domain revealed a surprisingly fragmented abundance pattern. We typically found highly conserved factors missing in individual lineages without any evidence for a methodological artefact. This passes the batton to the experimentalists to determine if, and to what extent, these factors are indeed involved in ribosome biogenesis. Using the late steps in large subunit maturation as an example, we provide evidence that archaea follow fundamentally similar but, at least in parts, highly simplified strategies to assemble their ribosomes. This underlines the role of archaea as potential model systems to elucidate general concepts of ribosome biogenesis and highlights evolutionary strategies to increase the fidelity of this process on the eukaryotic lineage.

## Data Availability Statement

Publicly available datasets were analyzed in this study. This data can be found at: https://applbio.biologie.uni-frankfurt.de/download/RibosomeBiogenesis.

## Author Contributions

IE conceived and planned the study and drafted the manuscript with help from MB. MTB and KB curated the data. MB, VT, and SS performed the analyses. IE, MB, MTB, and KB interpreted the results. All authors contributed to the article and approved the submitted version.

## Funding

This work was supported by the Research Funding Program Landes-Offensive zur Entwicklung Wissenschaftlich-ökonomischer Exzellenz (LOEWE) of the State of Hessen, Research Center for Translational Biodiversity Genomics (TBG) to IE, the Deutsche Forschungsgemeinschaft (SFB1190 to MTB, SFB860 to KB, and EB-285-2/2 to IE), and the University Medical Centre Göttingen (to MTB and KB).

## Conflict of Interest

The authors declare that the research was conducted in the absence of any commercial or financial relationships that could be construed as a potential conflict of interest.

## Publisher’s Note

All claims expressed in this article are solely those of the authors and do not necessarily represent those of their affiliated organizations, or those of the publisher, the editors and the reviewers. Any product that may be evaluated in this article, or claim that may be made by its manufacturer, is not guaranteed or endorsed by the publisher.

## Footnotes

^1^ https://www.ebi.ac.uk/reference_proteomes/

^2^ https://github.com/BIONF/fDog/

^3^ https://github.com/BIONF/FAS
