# Supplementary material for: Tracing Eukaryotic Ribosome Biogenesis Factors Into the Archaeal Domain Sheds Light on the Evolution of Functional Complexity
Source: Front Microbiol. 2021 Sep 16;12:739000. doi: 10.3389/fmicb.2021.739000 (PMC8481954; doi:10.3389/fmicb.2021.739000)
Supplement: Supplementary file 1 [file Data_Sheet_1.zip › Supplementary Figures and Tables S7-9.DOCX]

# Supplementary Information

## Supplementary Figures S1 - S23

**A** **B**


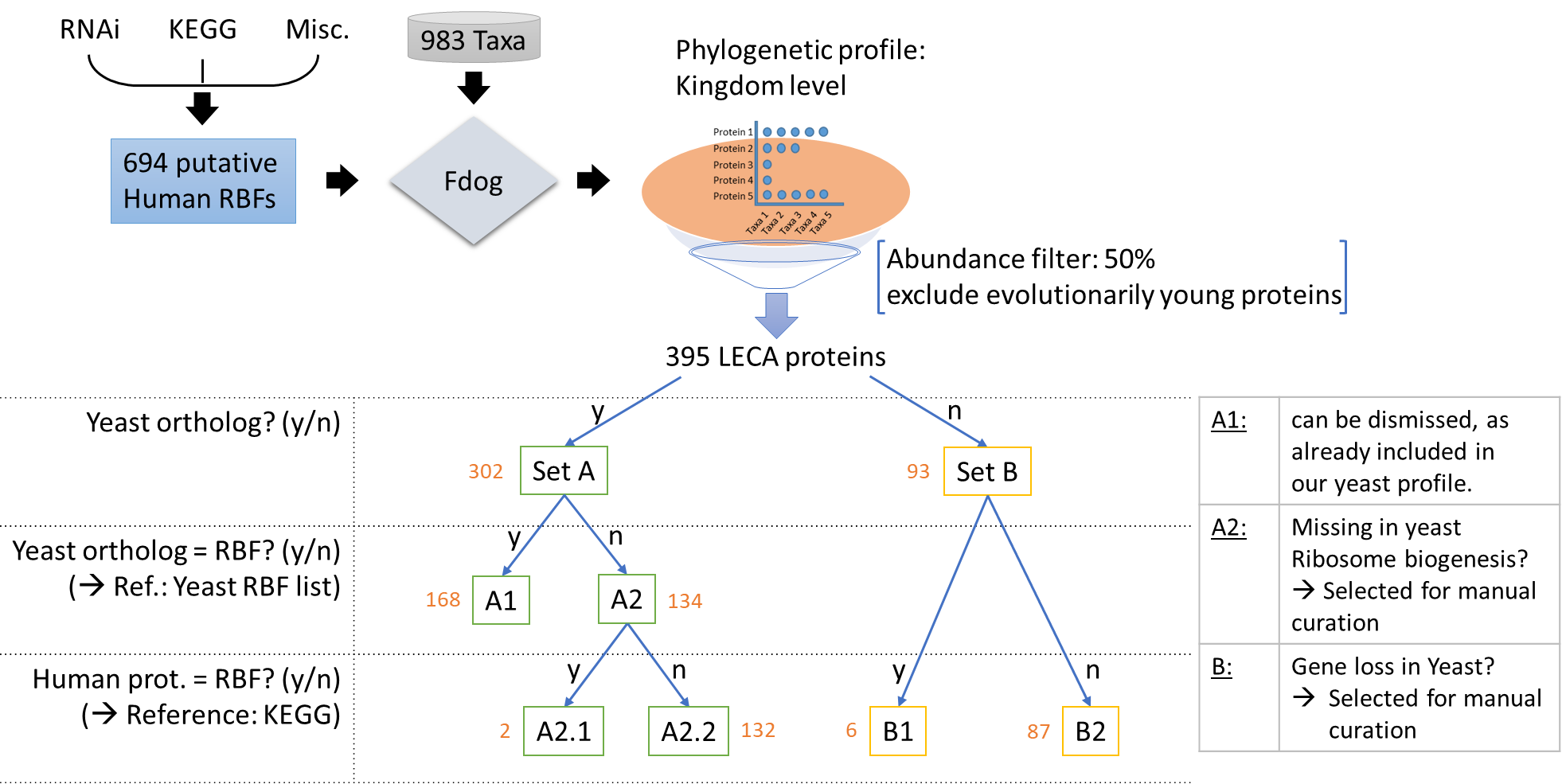

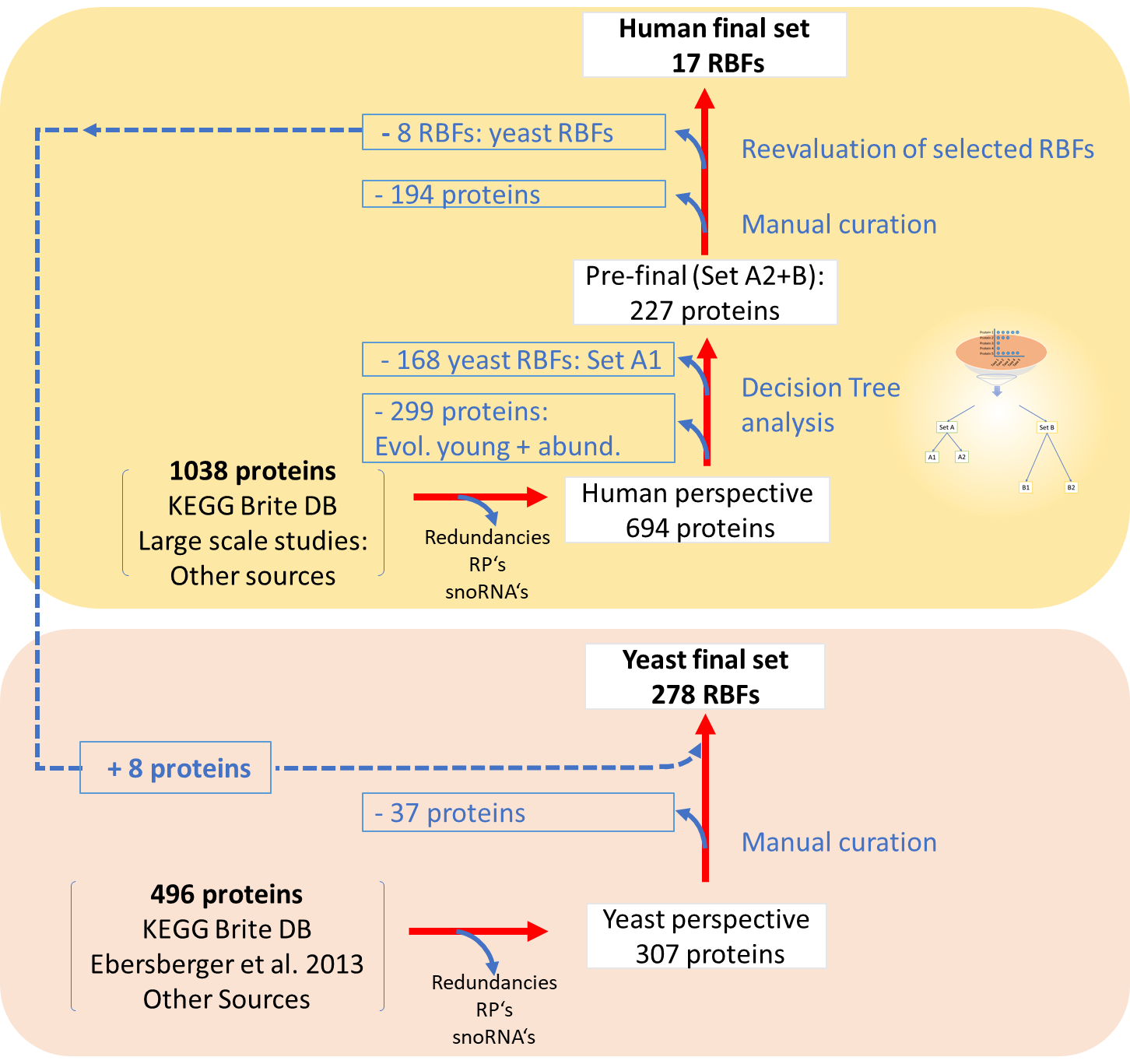


**Supplementary Figure S1. Workflow of the compilation and curation of the RBF_euk_ set.** (A) Workflow overview. (B) Details on the decision tree approach to select the final set of human RBFs to be included into the analysis


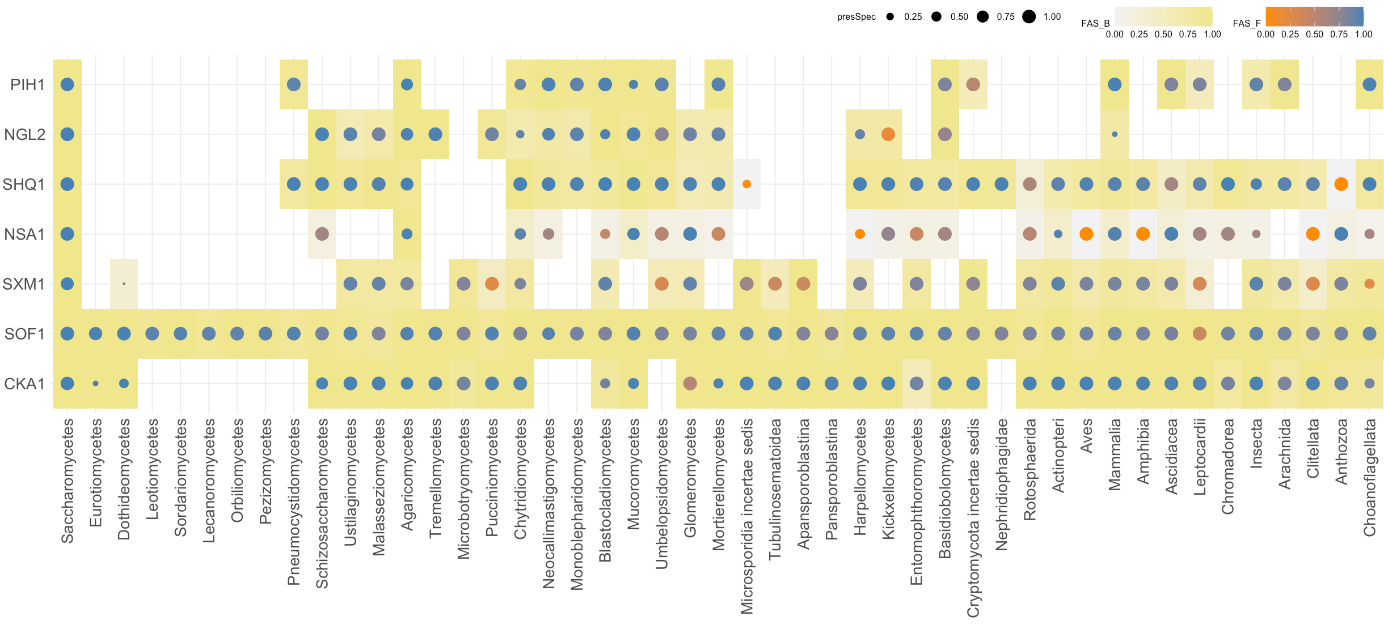


**Supplementary Figure S2. Yeast RBF loss within the Pezizomycotina. Phylogenetic profiles are subsumed on the class level.** Dot size reflects the fraction of species subsumed in each class harboring an ortholog to the yeast RBF. Dot color and cell color indicate similarity between the yeast protein and its ortholog using the yeast protein (dot) or the ortholog (cell) as reference. The taxa subsumed in the box jointly form the Pezizomycotina. Sof1 was added to allow the display of all taxa


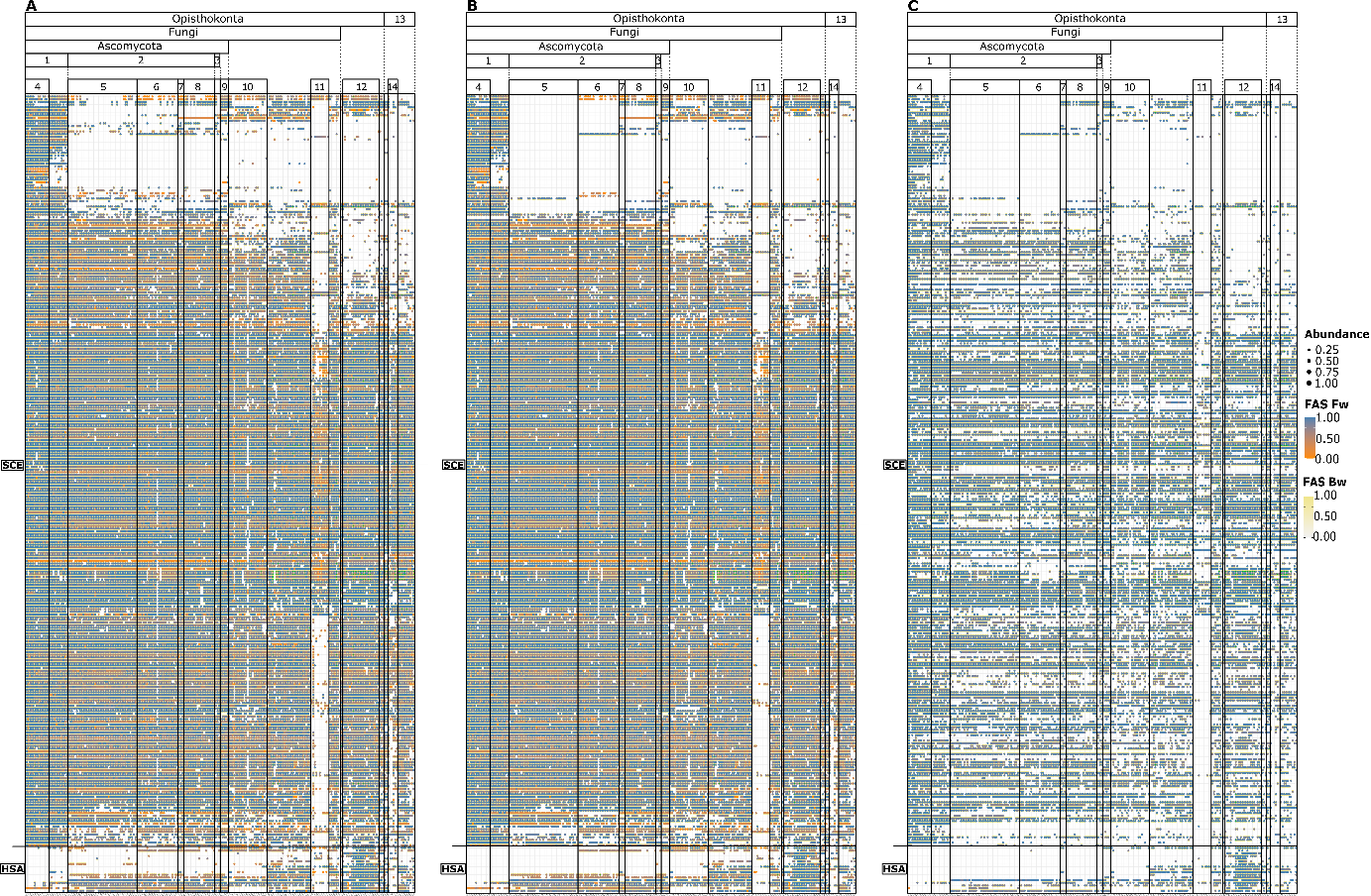


**Supplementary Figure S3. The effect of filtering on the RBF_euk_ dataset.** Shown is only the eukaryotic part of the phylogenetic profiles. SCE – members of the RBF_yeast_ set; HSA – members of the RBF_human_ set. (A) unfiltered set, (B) abundance filter on the class level. An ortholog was considered present only if it was represented in at least half of the species subsumed in a class. (C) The abundance filter was applied to the phylogenetic profile harboring only orthologs whose domain architectures are similar to the seed protein (Delta_FAS_ < 0.25). Taxonomic group labels: 1 – Saccharomycotina; 2 – Pezizomycotina; 3 – Taphrinomycotina; 4 – Saccharomycetceae; 5 – Dothideomycetes; 6 – Eurotiomycetes; 7 – Leotiomycetes; 8 – Eurotiomycetes; 9 – Schizosaccharomycetes; 10 – Basidiomycota; 11 – Microsporidia; 12 – Metazoa; 13 – Bikonta; 14 – Alveolata & Metamonada

PROSY MGKSNNKKKGN-------------KNLKSGGSPIKGG**K-LKAS**HILVKKLSLAQQICDDL

ASPFU MAPKNNAKGGDKKGKGKDAS--EGDKGKGGGKGLKPA**TSINVR**HILCEKFSKKEEALEKL

BATDJ MPPKKTGKAKP-QKQNE-----DSGDVAVKGGKLKAA**NSIKVR**HILCEKHSKIMEALELL

APIME MPPKKNTNTSK-ANKSKTV---ETNNGKEEKKKGNAG**NAIKVR**HILCEKQSKILEALEKL

CAEEL MPPKKTPKGGA-SGSGK-----DDGGGQKKEAK-GGG**TAVKVR**HILCEKQGKALEAIEKL

DROME MPPKKDAKSGKDAGKGGKK---PAAEDKSAGKEKKGG**NAVKVR**HILCEKQGKITEAMEKL

CHICK MAPKGKGG-GK-AGKGG-----ESGEGKAQGPK-GGG**SAVKVR**HILCEKHGRAMEAMEKL

HUMAN MPPKGKSGSGK-AGKGGAASGSDSADKKAQGPK-GGG**NAVKVR**HILCEKHGKIMEAMEKL

XENTR MPPKGKGGKGA---KGGAASG-EAADKKAQTPK--GG**NAVKVR**HILCEKHGKVMEAMEKL

* . .. ::. *** :* . : : *

PROSY TEGISFQELAKKYSECSSKNKGGNLGEFGKGKMVPEFWNACVKLKVGHISEP------VR

ASPFU RNGAKFDDVAREYSEDKAR-QGGSLGWKVRGSLNADFEKAAYELEPSTTANPKYVE--VK

BATDJ KSGQRFDKVAEQYSEDKAK-AGGSLGWMTRGSMVGVFQDAAFLLVPSTPDKPIYTNPPVK

APIME KAGGKFNEVAAIYSEDKAR-SGGDLGWMIKGSMVGPFQEAAFALPISSINSPIYTDPPIK

CAEEL KSGMKFNEVAAQYSEDKAR-SGGDLGWMTRGSMVGPFQDAAFALSNSSCDKPIYTDPPVK

DROME KAGQKFPEVAAAYSEDKAR-QGGDLGWQIRGAMVGPFQDAAFALPISTVNNPVYTDPPIK

CHICK KAGVRFSEVASQYSEDKAR-QGGDLGWMTRGSMVGPFQEAAFALPVSSMDKPVYTDPPVK

HUMAN KSGMRFNEVAAQYSEDKAR-QGGDLGWMTRGSMVGPFQEAAFALPVSGMDKPVFTDPPVK

XENTR KSGVRFSEVATQYSEDKAR-QGGDLGWMTRGSMVGPFQDAAFALPVSTMDKPVYTDPPVK

* * .:* *** .:. **.** .* : * .*. * . .* :.

PROSY SQFGYHIIKRTM--

ASPFU TGFGYHIIMVEGRK

BATDJ SNFGYHIIMVEDRK

APIME TKFGYHIIMVESKK

CAEEL TKFGYHVIMVEGKK

DROME TKFGYHIIMVEGKK

CHICK TKFGYHIIMVEGRK

HUMAN TKFGYHIIMVEGRK

XENTR TKFGYHIIMVEGRK

: ****:*

**Supplementary Figure S4. Multiple sequence alignment of animal and fungal and archaeal PIN4**. The box indicates the motif that was found essential for the association of animal PIN4 with the pre-rRNP complex (Fujiyama-Nakamura, et al. 2009). ASPFU: *Aspergillus fumigatus* - Q4WJM6; BATDJ: *Batrachochytrium dendrobatidis* - F4P849; HUMAN: *Homo sapiens* - Q9Y237; XENTR: *Xenopus tropicalis* - Q6P4K8; CHICK: *Gallus gallus* - F1NCD7; CAEEL: *Caenorhabditis elegans* - Q9NAF9; DROME: *Drosophila melanogaster* - Q9VBU4; CULQU: *Culex quinquefasciatus* - B0X1I8; APIME: *Apis mellifera* - A0A7M7L0Q4; PROSY: *Candidatus Prometheoarchaeum syntrophicum* - WP_147661883.1

**Supplementary Figure S5. Domain architectures of yeast RNase MRP components whose functional equivalent in humans is not detected as an ortholog.** Four yeast proteins have a traceability index below 0.75 in humans: Snm1=0,58; Pop6=0,48; Pop8=0,58; Rmp1=0. Pfam Domain Ids: Alba - PF01918; Rpp20 - PF01918; Rpr2 - PF04032; RNase_P_Pop3 - PF08228

**Supplementary Figure S6. PA2G4 does not identify Arx1 as its best blast hit.** Both yeast Arx1 and human PA2G4 share the presence of a Peptidase_M24 domain, however PA2G4 does not contain the extensive C-terminal region present in Arx1. BlastP indicates the bit score and the percent identity to yeast Arx1 and Map2, respectively, using PA2G4 as a query. Species abbreviations: Sce – Saccharomyces cerevisiae; Hsa – Homo sapiens

**Figure S7. Maxmimum likelihood phylogeny of orthologs to Arx1 and PA2G4 in the Ascomycota and Basidiomycota.** Orthologs of PA2G4 and Arx1 in Taphrinomycotina and Saccharomycotina are shaded in green and grey respectively. They form two distinct clades, which most likely arose by a gene duplication event. Branch lengths are drawn to scale and are given in substitutions per site. They indicate an accelerated substitution rate of both PA2G4 and Arx1 orthologs in Taphrinomycotina and Saccharomycotina relative to the corresponding proteins in other fungi. Arx1 of *Saccharomyces cerevisiae* is highlighted in yellow.


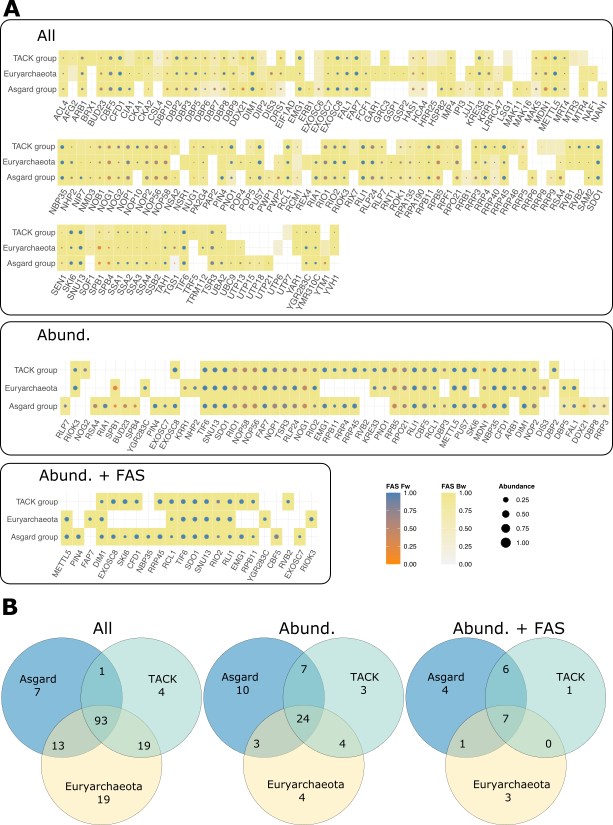


**Supplementary Figure S8. Representation of eukaryotic ribosome biogenesis factors in the archaeal domain.** (A) Phylogenetic profiles of the RBFeuk in the major archaeal clades. (B) The distribution of RBF orthologs among the three archaeal groups. All – unfiltered; Abund. – only RBFs with orthologs identified in at least half of the subsumed taxa are shown; Abund. + FAS – Orthologs with a FAS score below 0.75 were removed prior to the application of the abundance filter. The data underlying this figure including the data for the DPANN group that is not shown here because of its non-representative taxon sampling are deposited in supplementary table S10.

**A**

**B**


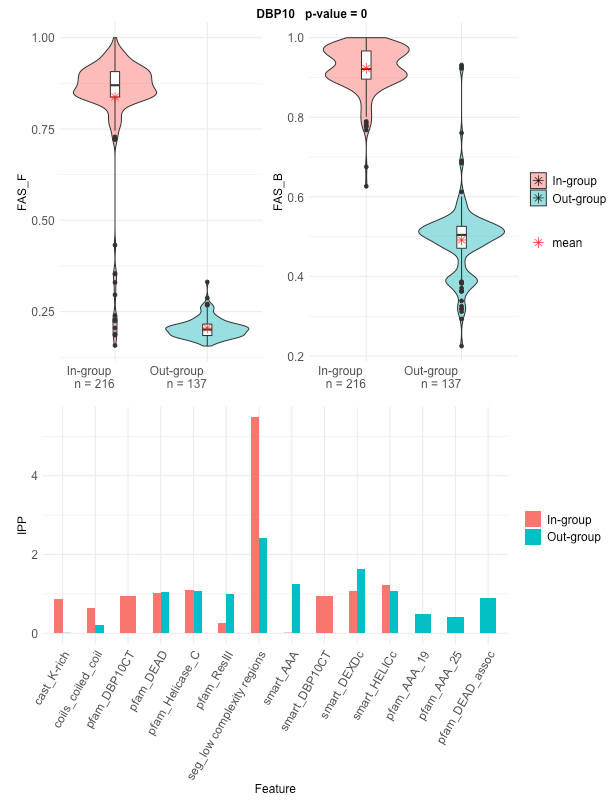

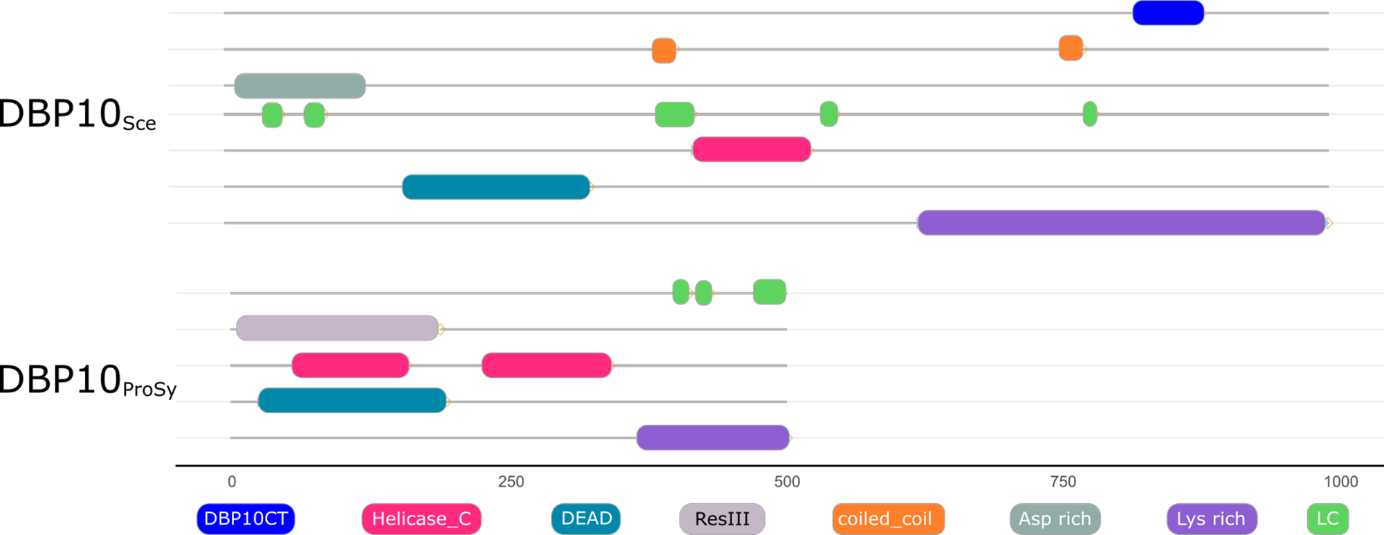


**Supplementary Figure S9. Group comparison of eukaryotic and archaeal orthologs to Dbp10yeast.** (A) Domain architectures of Dbp10_yeast_ with its ortholog in *Candidatus Prometheoarchaeum syntrophicum*. (B) The violin plots show the distribution of domain architecture similarity scores between eukaryotic orthologs (In-group) and archaeal orthologs (Out-group) of Dbp10 in yeast. FAS_F – Domain architecture scores are computed using the yeast protein as a reference, i.e. yeast domains absent in the ortholog reduce the score, but not vice versa. FAS_R Domain architecture scores are computed using the ortholog as a reference, i.e. domains present in the ortholog but not in the yeast protein reduce the score, but not vice versa. The bar plot denotes the average instances per protein for each domain in In-group and Out-group orthologs, respectively. Overlapping Pfam domains are considered.


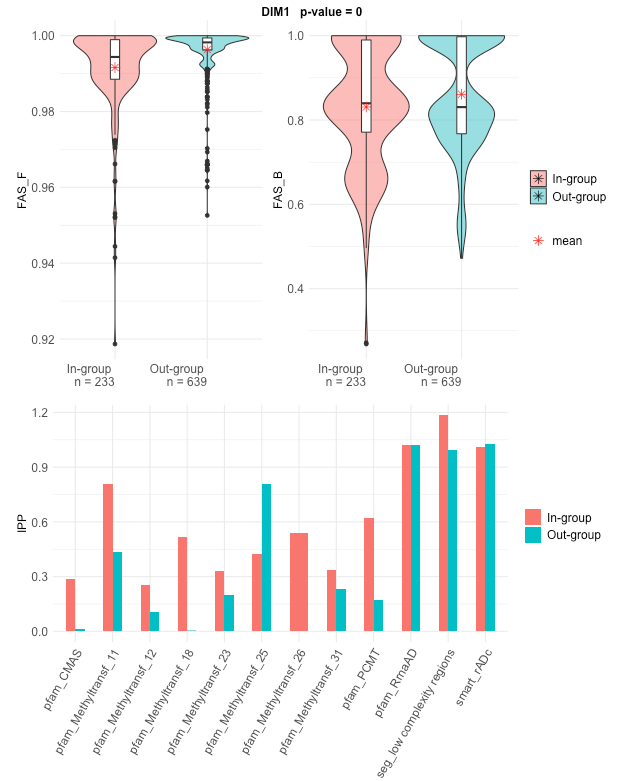


**Supplementary Figure S10. Group comparison of eukaryotic and archaeal orthologs to Dim1_yeast_.** The violin plots show the distribution of domain architecture similarity scores between eukaryotic orthologs (In-group) and archaeal orthologs (Out-group) of Dim1 in yeast. FAS_F – Domain architecture scores are computed using the yeast protein as a reference, i.e. yeast domains absent in the ortholog reduce the score, but not vice versa. FAS_R Domain architecture scores are computed using the ortholog as a reference, i.e. domains present in the ortholog but not in the yeast protein reduce the score, but not vice versa. The bar plot denotes the average instances per protein for each domain in In-group and Out-group orthologs, respectively. Overlapping Pfam domains are considered. See Supplementary Figure S9 for the domain architecture visualization

A


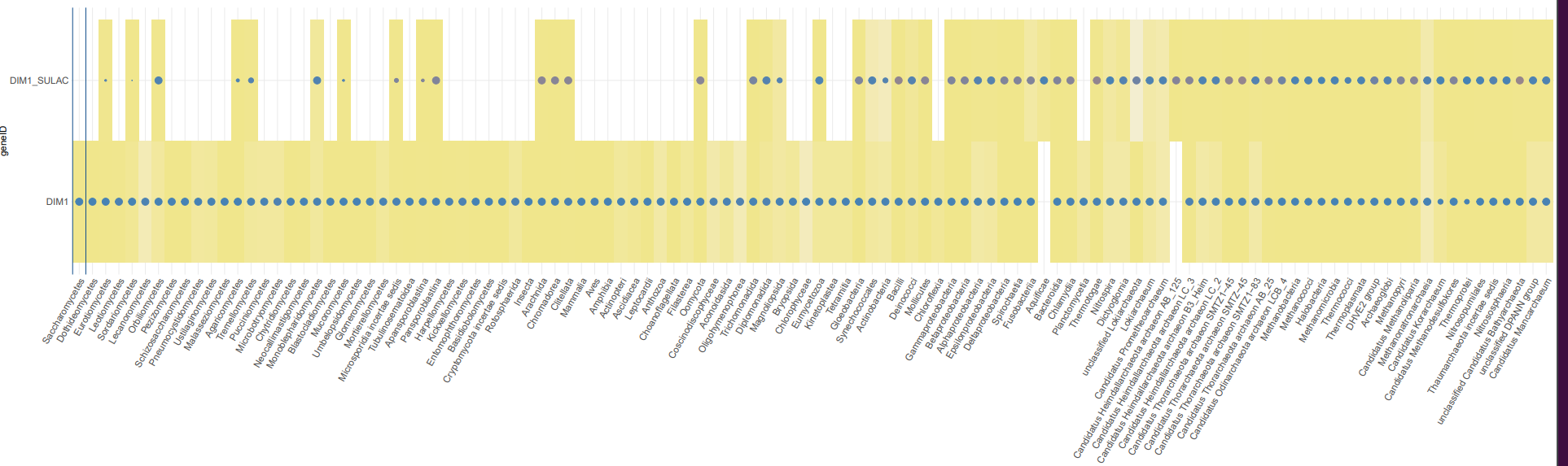


B


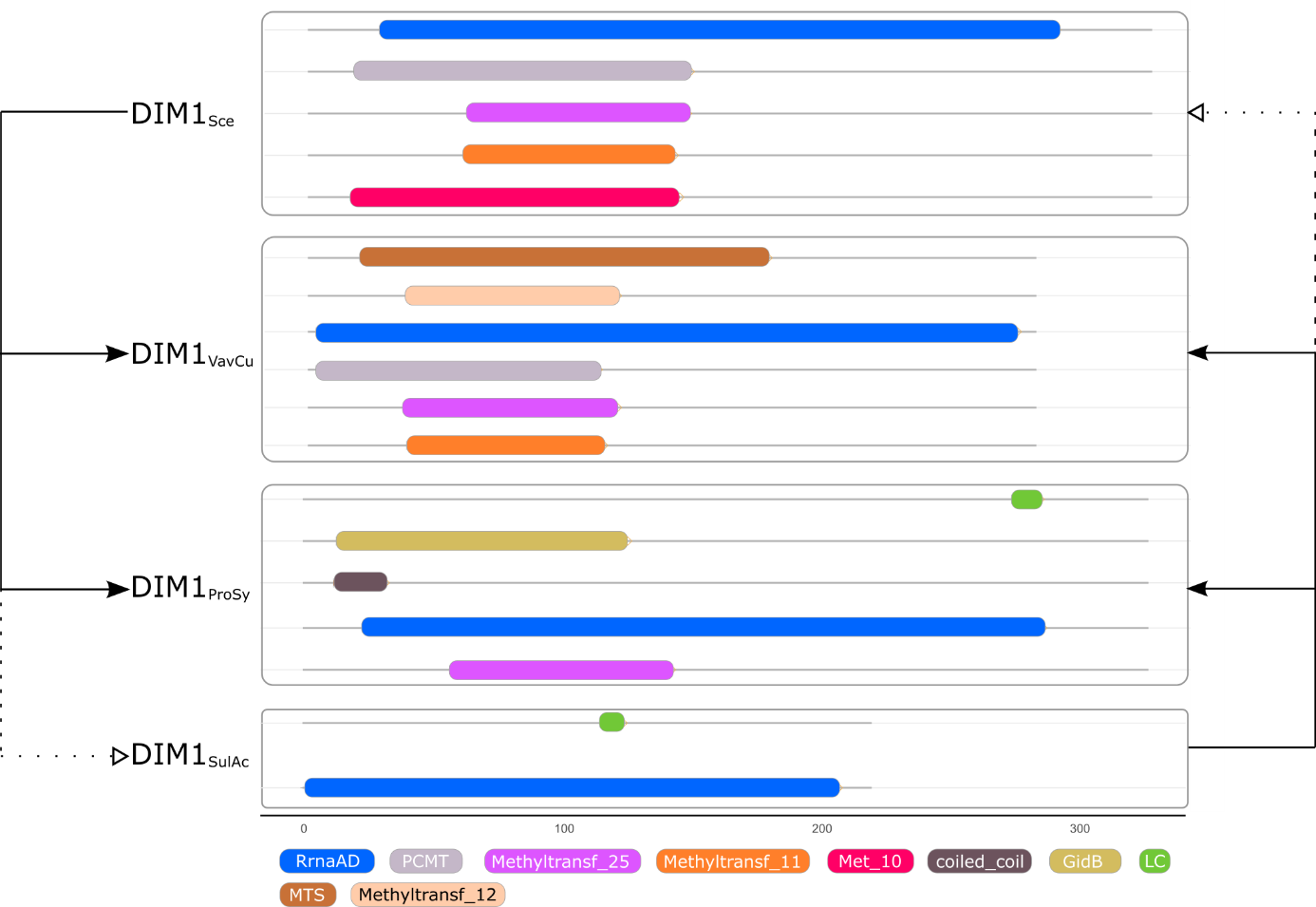


**Supplementary Figure S11. The stepping-stone approach reveals that Dim1 is consistently present in the archaea.** A) Phylogenetic profiles of *S. acidocaldarius* Dim1 (top) and of yeast Dim1 (bottom). B) Domain architecture comparison of selected orthologs of Dim1_yeast_, Dim1_VavCu_, Dim1_ProSy_ and Dim1_SulAc_. Arrows indicate direction of ortholog search. Dashed lines indicate that the proteins do not identify each other as a BlastP hit. All proteins share the presence of the characteristic Ribsomal RNA Adenine Dimethylase domain (RrnaAD; Pfam Id PF00398). Species abbreviations: Sce – *Saccharomyces cerevisiae*; VavCu – *Vavraia culicis* (Microsporidia); ProSy – *Candidatus Prometheoarchaeum syntrophicum*; SulAc – *Sulfulobus acidocaldarius*

**Supplementary Figure S12. Length distribution of Dim1 orthologs.** Dim1 in Sulfolobales (left); Dim1 in other archaeal, eukaryotic and bacterial taxa (right)

**Supplementary Figure S13. Phylogenetic profiles of KRE33, RIO2, and EMG1 seeded with a yeast and an archaeal protein, respectively.**


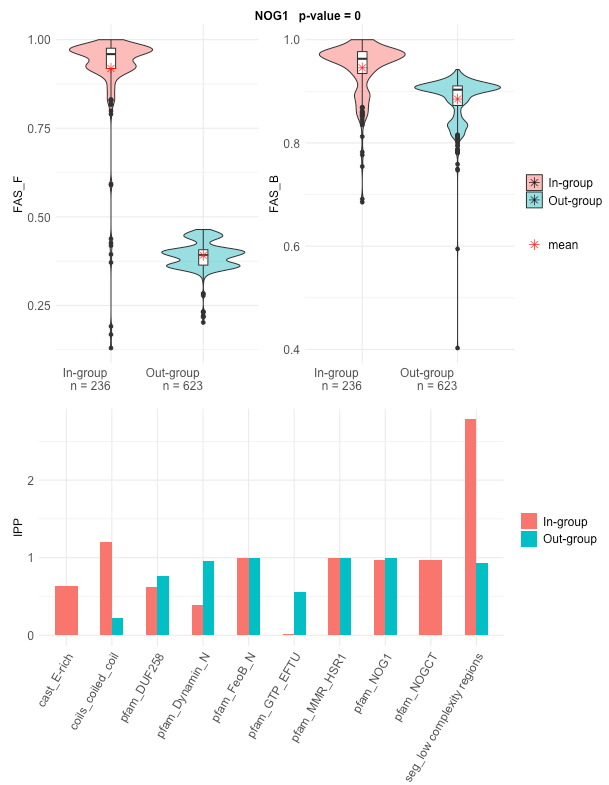


**Supplementary Figure S14. Group comparison of eukaryotic and archaeal orthologs to Nog1yeast.** The violin plots show the distribution of domain architecture similarity scores between eukaryotic orthologs (In-group) and archaeal orthologs (Out-group) of Nog1 in yeast. FAS_F – Domain architecture scores are computed using the yeast protein as a reference, i.e. yeast domains absent in the ortholog reduce the score, but not vice versa. FAS_R Domain architecture scores are computed using the ortholog as a reference, i.e. domains present in the ortholog but not in the yeast protein reduce the score, but not vice versa. The bar plot denotes the average instances per protein for each domain in In-group and Out-group orthologs, respectively, considering also overlapping Pfam domains. Note that the NOGCT domain (Pfam Id PF08155) and the E-rich region is confined to the eukaryotic representatives of Nog1 (see main text and Figure 8C).

**Supplementary Figure S15. The numbers of proteins per taxonomic group harboring a Nog1 Pfam domain.** The total number of analyzed gene sets per taxonomic group is given in parenthesis. The distribution was truncated at 10. This removes 2 animal and 11 plant species with more than 10 proteins harboring a Nog1 Pfam domain from the plot. Nog1 – Pfam Id PF06858

**Supplementary Figure S16. The number of proteins per taxon harboring a Ribosomal_L24e Pfam domain**. The total number of analyzed gene sets per taxonomic group is given in parenthesis. The distribution was truncated at 10. This removes 6 animal and 19 plant species with more than 10 proteins harboring a Ribosomal_L24e Pfam domain from the plot. Ribosomal_L24e – Pfam Id PF01246.

**Supplementary Figure S17. The number of proteins per taxon harboring a Ribosomal_L10 Pfam domain**. The total number of analyzed gene sets per taxonomic group is given in parenthesis. The distribution was truncated at 10. This removes 6 animal and 20 plant species with more than 10 proteins harboring a Ribosomal_L10 Pfam domain from the plot. Ribosomal_L10 – Pfam Id PF00466.


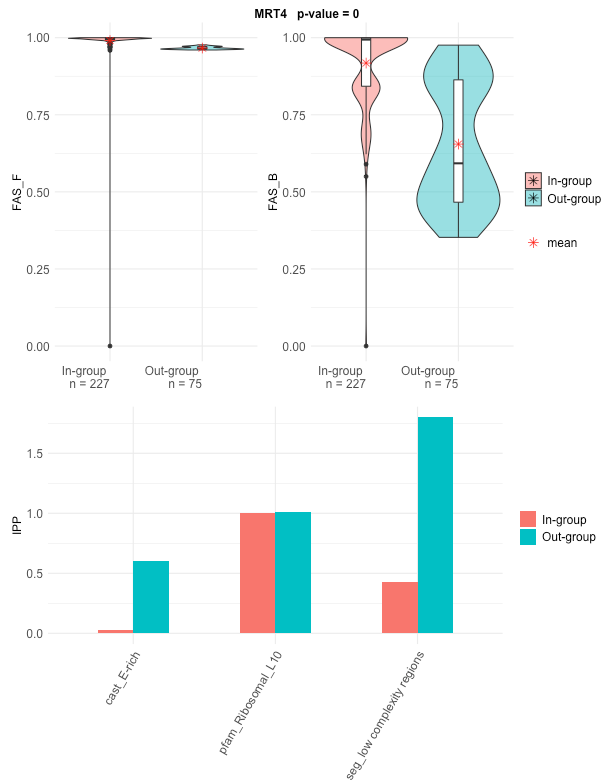


**Supplementary Figure S18. Group comparison of eukaryotic and archaeal orthologs to Mrt4.** The violin plots show the distribution of domain architecture similarity scores between eukaryotic orthologs (In-group) and archaeal orthologs (Out-group) of Mrt4 in yeast. FAS_F – Domain architecture scores are computed using the yeast protein as a reference, i.e. yeast domains absent in the ortholog reduce the score, but not vice versa. FAS_R Domain architecture scores are computed using the ortholog as a reference, i.e. domains present in the ortholog but not in the yeast protein reduce the score, but not vice versa. The bar plot denotes the average instances per protein for each domain in In-group and Out-group orthologs, respectively. More than half of the archaeal orthologs harbor an E-rich domain (IPP=0.6)


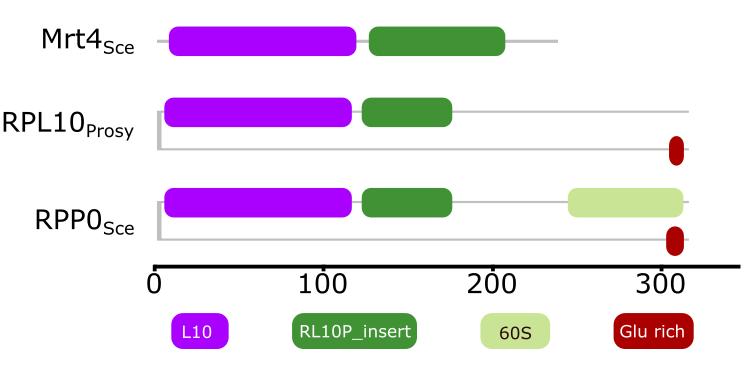


**Supplementary Figure S19. Domain architecture comparisons between the yeast proteins Mrt4, RPP0, and RPL10 of Candidatus Prometheoarchaeum syntrophicum.** L10 – Ribosomal_L10 (Pfam Id PF00466); 60S – Ribosomal_60s (Pfam Id PF00428); RL10P_insert (Pfam Id PF17777). Mrt4 – Uniprot Id P33201; RPP0 – P05317; RPL10_Prosy_ – NCBI Id WP_147662854. Sce – *Saccharomyces cerevisiae*; Prosy – *Candidatus Prometheoarchaeum syntrophicum*

**Supplementary Figure S20. The number of proteins per taxon harboring a Nmd3 Pfam domain**. Most of the analyzed species carry only a single protein with an Nmd3 Pfam domain. The total number of analyzed gene sets per taxonomic group is given in parenthesis. Only 54 of the 858 archaeal taxa lack a protein with an Nmd3 domain, indicating that Nmd3 is prevalent in the archaeal domain. Nmd3 – Pfam Id PF04981.

A


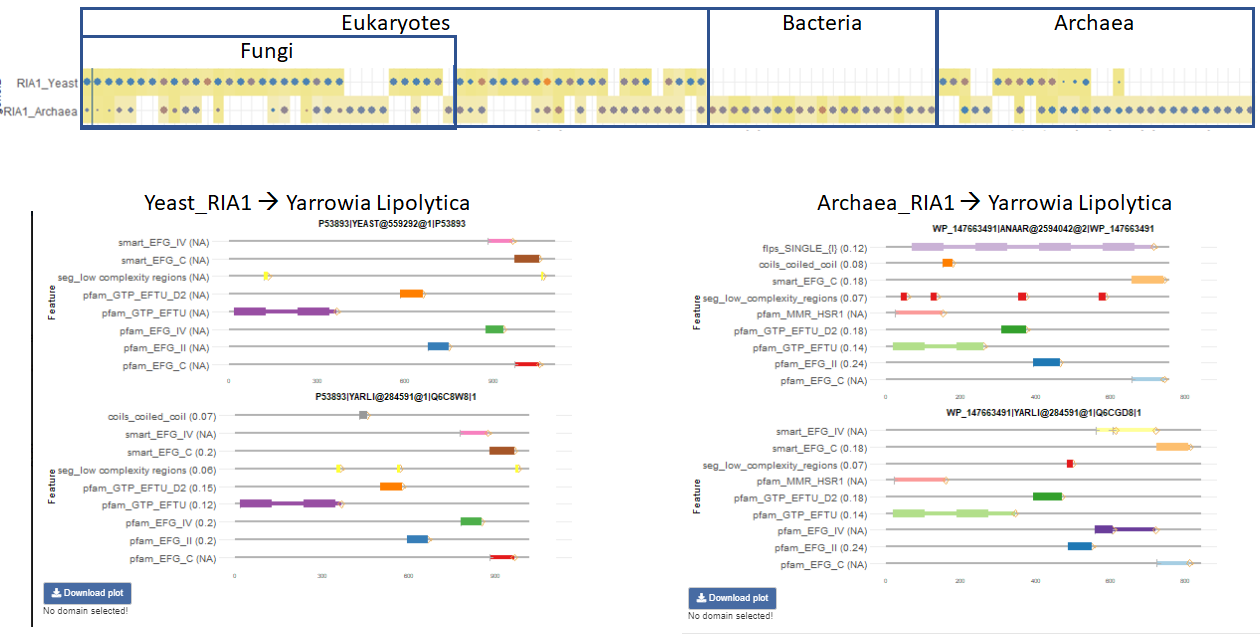


B

**Supplementary Figure S21. Comparison of yeast Ria1/Efl1 and its counterparts in the archaea.** (A) Phylogenetic profiles of yeast Ria1 and its ortholog in *Candidatus Prometheoarchaeum syntrophicum*, respectively. (B) Domain architecture comparisons between yeast RIA1 (top) and its ortholog in *Candidatus Prometheoarchaeum syntrophicum*
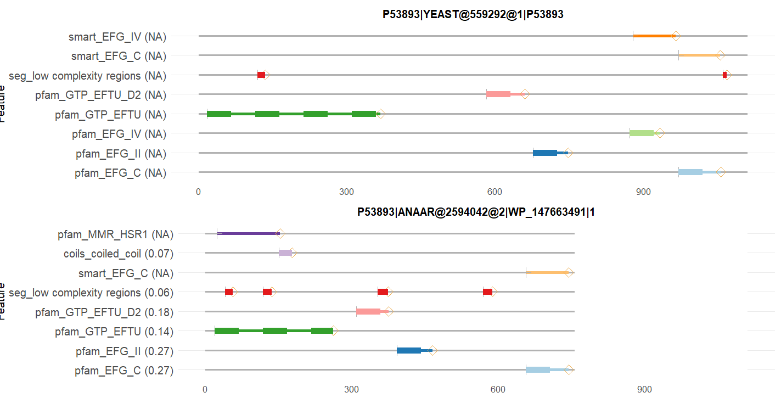


**A**


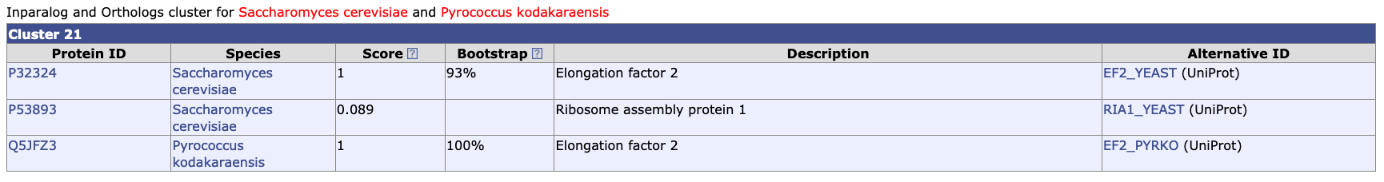


**B**

**RIA1_yeast_ vs RIA1_yli_ EF2_psy_ vs. EF2_yli_**


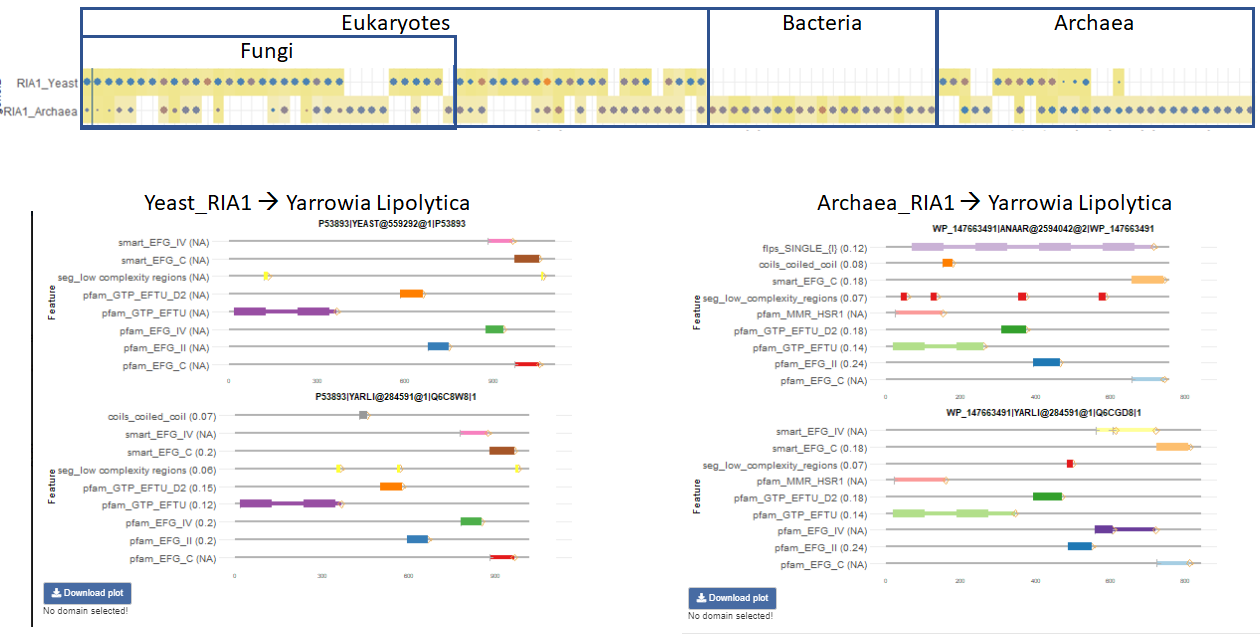


**Supplementary Figure S22. RIA1_yeast_ and EF2_yeast_ are ancient paralogs that identify the same ortholog in archaea.** (A) InParanoid Cluster 21 identifies RIA1_yeast_ (Uniprot Id P53893) and EF2_yeast_ as co-orthologs to EF2 of the archaeum *Pyrococcus kodakarensis* (Uniprot Id Q5JFZ3). (B) Domain architecture comparisons RIA1_yeast_ and RIA1_yli_ (Uniprot Id Q6C8W8)(left), and of the archaeal RIA1 ortholog (NCBI Id WP_147663491; cf. Supplementary Figure S18) against EF2_yli_ that is found when using the archaeal protein as a seed (right). Species abbreviations: yeast – *Saccharomyces cerevisiae* (Saccharomycestes); yli – *Yarrowia lipolytica* (Saccharomycetes); psy – *Candidatus Prometheoarchaeum syntrophicum* (Asgard group)

**A**


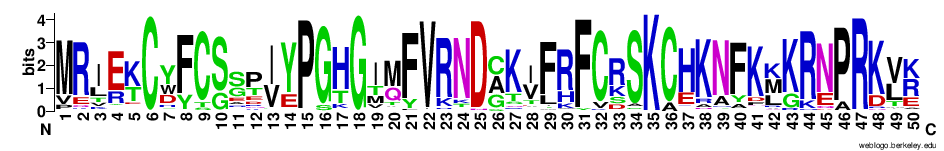
**B**


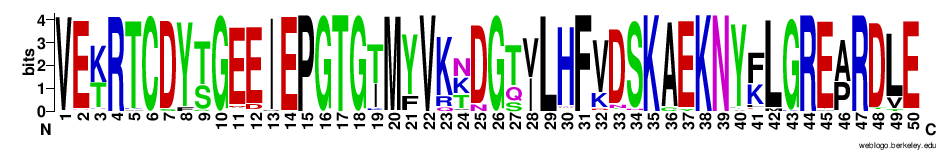


**Supplementary figure S23.** **Sequence logo representing the the N-terminal 50 alignment columns of Rlp24 orthologs.** (A) Sequence logo of all Rlp24 orthologs detected in this study. The arrows indicate the conserved cysteine residues in eukaryotic Rlp24 and in most archaeal L24e (Saveanu, et al. 2003). (B) Sequence logo of the subset of archaeal L24e proteins from Halobacteria and Thermoplasmata whose domain architecture resembles that of the eukaryotic Rlp24 (cf. Figure 8D in main text). Three of the four cysteine residues are not conserved.

## Supplementary Tables S1-S9

**Supplementary Table S1. Data Compilation: List of 307 Yeast proteins**

See file Birikmen_ SupplTabs1-6_10.xlsx

**Supplementary Table S2. Data Compilation: List of 695 Human proteins**

See file Birikmen_ SupplTabs1-6_10.xlsx

**Supplementary Table S3. List of taxa analysed in this study**

See file Birikmen_ SupplTabs1-6_10.xlsx

**Supplementary Table S4. Decision tree analysis and final set**

See file Birikmen_ SupplTabs1-6_10.xlsx

**Supplementary Table S5. Phylostratigraphy of 303 RBFs**

See file Birikmen_ SupplTabs1-6_10.xlsx

**Supplementary Table S6. Traceability indices of yeast RBFs**

See file Birikmen_ SupplTabs1-6_10.xlsx

**Supplementary Table S7. Yeast RBFs present in at least 85% of the analyzed archaeal taxa**

| RBF^1^ | Description | | Site of action |
| --- | --- | --- | --- |
| RLP24 | RBF acting as Placeholder, intertwined by NOG1 C-Terminal tail. | - | Pre-60S |
| SDO1 | Ribosome maturation protein SDO1 | PTC-maturation |  |
| TIF6 | Eukaryotic translation initiation factor 6 (eIF-6) |  |  |
| RIO1 | Serine/threonine-protein kinase RIO1 (EC 2.7.11.1) (EC 3.6.3.-) (Ribosomal RNA-processing protein 10) | - | Pre-40S |
| FAP7 | Adenylate kinase isoenzyme 6 homolog FAP7 (AK6) (EC 2.7.4.3) (Dual activity adenylate kinase/ATPase) (AK/ATPase) (POS9-activating factor 7) | - |  |
| PUS7 | Pseudouridine synthase, (Ψ50) in the 5S rRNA, which is introduced by the pseudouridine synthetase Pus7 that specifically modifies targets with the RSUNΨAR. | - | rRNA-processing |
| METTL5 | m6A methyltransferase, methylates the 16/18S rRNA | Methyl-transferase |  |
| NOP1 | rRNA 2'-O-methyltransferase fibrillarin | Box C/D RNP |  |
| NOP56 | Nucleolar protein 56 |  |  |
| NOP58 | Nucleolar protein 58 |  |  |
| SNU13 | 13 kDa ribonucleoprotein-associated protein (Small nuclear ribonucleoprotein-associated protein 1) |  |  |
| CBF5 | H/ACA ribonucleoprotein complex subunit CBF5 (EC 5.4.99.-) (Centromere-binding factor 5) (Centromere/microtubule-binding protein CBF5) (H/ACA snoRNP protein CBF5) (Small nucleolar RNP protein CBF5) (p64') | Box H/ACA RNP |  |
| CFD1 | Cytosolic Fe-S cluster assembly factor CFD1 (Cytosolic Fe-S cluster-deficient protein 1) (Ribosomal export protein 19) | Fe-S cluster biogenesis | other |
| NPB35 | Cytosolic Fe-S cluster assembly factor NBP35 (Nucleotide-binding protein 35) |  |  |
| RLI1 | Translation initiation factor RLI1 (ATP-binding cassette sub-family E member RLI1) (RNase L inhibitor) | - |  |
| RPB5^1^ | DNA-directed RNA polymerases I, II, and III subunit RPABC1 | RNA polymerase subunits |  |
| RPO21^1^ | DNA-directed RNA polymerase II subunit RPB1 |  |  |

1 The two subunits of the RNA polymerase II serve as positive control and are not RBFs in the strict sense

**Supplementary Table S8. Yeast RBFs with missing orthologs in the microsporidia.**

| RBF | Ti^1^ | RBF | Ti^1^ | RBF | Ti^1^ |
| --- | --- | --- | --- | --- | --- |
| ROK1 | 0,99 | NOP9 | 0,87 | KAP104 | 0,66 |
| REX2 | 0,99 | CIC1 | 0,87 | KAP114 | 0,62 |
| RIA1 | 0,98 | NOP13 | 0,87 | NOP6 | 0,57 |
| PSE1 | 0,97 | POP4 | 0,87 | NOP19 | 0,54 |
| BMT2 | 0,97 | RAI1 | 0,87 | SNM1 | 0,50 |
| BMT6 | 0,97 | NAF1 | 0,86 | CGR1 | 0,44 |
| YMR310C | 0,96 | TMA16 | 0,85 | RSA3 | 0,43 |
| PIH1 | 0,95 | POP7 | 0,85 | RKM2 | 0,40 |
| LTV1 | 0,95 | UTP5 | 0,85 | POP6 | 0,40 |
| AFG2 | 0,95 | CMS1 | 0,84 | URB2 | 0,38 |
| URB1 | 0,94 | NOP53 | 0,84 | RIX1 | 0,37 |
| RRP12 | 0,94 | BCD1 | 0,84 | BUD21 | 0,37 |
| MAK11 | 0,94 | LCP5 | 0,80 | NSA1 | 0,31 |
| UTP20 | 0,94 | KAP123 | 0,80 | SLX9 | 0,26 |
| RIX7 | 0,94 | MTR3 | 0,80 | NAN1 | 0,07 |
| BFR2 | 0,93 | IPI1 | 0,78 | SYO1 | 0,01 |
| RRP42 | 0,92 | ACL4 | 0,76 | NOP8 | 0,00 |
| BCP1 | 0,92 | RRP43 | 0,75 | UTP9 | 0,00 |
| SQT1 | 0,91 | MTR2 | 0,75 | LOC1 | 0,00 |
| YVH1 | 0,90 | MEX67 | 0,75 | RSA1 | 0,00 |
| NGL2 | 0,89 | ALB1 | 0,74 | FAF1 | 0,00 |
| UTP8 | 0,89 | HIT1 | 0,74 | TMA23 | 0,00 |
| RRP36 | 0,89 | RRP15 | 0,74 | RMP1 | 0,00 |
| HCR1 | 0,89 | LRP1 | 0,74 | POP8 | 0,00 |
| RRP14 | 0,89 | ARX1 | 0,73 |  |  |
| POP5 | 0,89 | ECM1 | 0,72 |  |  |
| RRP17 | 0,88 | FYV7 | 0,72 |  |  |
| FCF2 | 0,88 | POP3 | 0,71 |  |  |
| NOP16 | 0,88 | NPL3 | 0,71 |  |  |
| NUG1 | 0,88 | PXR1 | 0,71 |  |  |
| RRP7 | 0,88 | YGR283C | 0,70 |  |  |
| UTP10 | 0,87 | MPP6 | 0,69 |  |  |

^1^Ti – Traceability index of the respective protein in *Antonospora locustae*. Values below 0.75 indicate that orthologs might be too diverged to warrant their identification

**Supplementary Table S9. Number of orthologs to eukaryotic RBFs in the top 20 archaeal taxa**

| **strain** | **ncbiID** | **# of orthologs** |
| --- | --- | --- |
| Candidatus_Prometheoarchaeum_syntrophicum | ncbi2594042 | 67 |
| Candidatus_Heimdallarchaeota_archaeon_B3_Heim | ncbi2012493 | 63 |
| Candidatus_Heimdallarchaeota_archaeon_LC_3 | ncbi1841598 | 61 |
| Pyrolobus_fumarii_1A | ncbi694429 | 59 |
| Pyrococcus_yayanosii_CH1 | ncbi529709 | 59 |
| Staphylothermus_hellenicus_DSM_12710 | ncbi591019 | 57 |
| Methanopyrus_sp._SNP6 | ncbi1937005 | 57 |
| Pyrodictium_occultum | ncbi2309 | 56 |
| Haloferax_sp._CBA1150 | ncbi2650754 | 56 |
| Ferroglobus_placidus_DSM_10642 | ncbi589924 | 56 |
| Pyrococcus_sp._NA2 | ncbi342949 | 55 |
| Pyrococcus_furiosus_DSM_3638 | ncbi186497 | 55 |
| Pyrococcus_furiosus_COM1 | ncbi1185654 | 55 |
| Pyrobaculum_aerophilum_str._IM2 | ncbi178306 | 55 |
| Methanopyrus_sp._KOL6 | ncbi1937004 | 55 |
| Hyperthermus_butylicus_DSM_5456 | ncbi415426 | 55 |
| Haloferax_sp._MBLA0078 | ncbi2666143 | 55 |
| Haloferax_sp._KTX1 | ncbi2600597 | 55 |
| Haloferax_larsenii_JCM_13917 | ncbi1227460 | 55 |
| Thermosphaera_aggregans_DSM_11486 | ncbi633148 | 54 |

**Supplementary Table S10. RBF repertoires in the three main archaeal lineages, TACK, Asgard, and Euryarchaeota**

See file Birikmen_ SupplTabs1-6_10.xlsx

**References**

Fujiyama-Nakamura S, et al. 2009. Parvulin (Par14), a peptidyl-prolyl cis-trans isomerase, is a novel rRNA processing factor that evolved in the metazoan lineage. Mol Cell Proteomics 8: 1552-1565. doi: 10.1074/mcp.M900147-MCP200

Saveanu C, et al. 2003. Sequential protein association with nascent 60S ribosomal particles. Mol Cell Biol 23: 4449-4460.
